# Nanopore-based consensus sequencing enables accurate multimodal tumor cell-free DNA profiling

**DOI:** 10.1101/gr.279144.124

**Published:** 2025-04

**Authors:** Li-Ting Chen, Myrthe Jager, Dàmi Rebergen, Geertruid J. Brink, Tom van den Ende, Willem Vanderlinden, Pauline Kolbeck, Marc Pagès-Gallego, Ymke van der Pol, Nicolle Besselink, Norbert Moldovan, Nizar Hami, Wigard P. Kloosterman, Hanneke van Laarhoven, Florent Mouliere, Ronald Zweemer, Jan Lipfert, Sarah Derks, Alessio Marcozzi, Jeroen de Ridder

**Affiliations:** 1 Center for Molecular Medicine University Medical Center Utrecht, Utrecht University, 3584 CX Utrecht, The Netherlands;; 2 Oncode Institute, 3521 AL Utrecht, The Netherlands;; 3 Cyclomics, 3584 CG Utrecht, The Netherlands;; 4 Department of Gynecologic Oncology, University Medical Center Utrecht, Utrecht University, 3584 CX Utrecht, The Netherlands;; 5 Department of Medical Oncology, Amsterdam UMC, University of Amsterdam, 1105 AZ, Amsterdam, The Netherlands;; 6 Cancer Center Amsterdam, Imaging and Biomarkers, 1105 AZ, Amsterdam, The Netherlands;; 7 Soft Condensed Matter and Biophysics, Department of Physics and Debye Institute for Nanomaterials Science, Utrecht University, 3584 CC Utrecht, The Netherlands;; 8 School of Physics and Astronomy, University of Edinburgh, EH9 3FD Edinburgh, United Kingdom;; 9 Department of Physics and Center for NanoScience, LMU Munich, 80799 Munich, Germany;; 10 Department of Pathology, Cancer Centre Amsterdam, Amsterdam UMC, Vrije Universiteit Amsterdam, 1105 AZ, Amsterdam, The Netherlands;; 11 Cancer Research UK National Biomarker Centre, University of Manchester, Manchester M20 4BX, United Kingdom

## Abstract

Shallow genome-wide cell-free DNA sequencing holds great promise for noninvasive cancer monitoring by providing reliable copy number alteration (CNA) and fragmentomic profiles. Single-nucleotide variations (SNVs) are, however, much harder to identify with low sequencing depth due to sequencing errors. Here, we present Nanopore Rolling Circle Amplification (RCA)-enhanced Consensus Sequencing (NanoRCS), which leverages RCA and consensus calling based on genome-wide long-read nanopore sequencing to enable simultaneous multimodal tumor fraction (TF) estimation through SNVs, CNAs, and fragmentomics. The efficacy of NanoRCS is tested on 18 cancer patient samples and seven healthy controls, demonstrating its ability to reliably detect TFs as low as 0.24%. In vitro experiments confirm that SNV measurements are essential for detecting TFs below 3%. NanoRCS provides an opportunity for cost-effective and rapid sample processing, which aligns well with clinical needs, particularly in settings where quick and accurate cancer monitoring is essential for personalized treatment strategies.

A recent advancement in cancer diagnostics involves the analysis of short cell-free DNA (cfDNA) molecules found in blood and various other bodily fluids (Wan et al. 2017). These molecules are primarily released by cells undergoing apoptosis and necrosis (Diaz and Bardelli 2014). In cancer patients, a fraction of the cfDNA stems from the tumor (circulating tumor DNA, ctDNA). Because these ctDNA molecules carry the genetic features of the tumor (Stroun et al. 1987; Nawroz et al. 1996; Husain et al. 2017; Burnham et al. 2018; McEwen et al. 2020), interrogating them offers exciting opportunities for minimally invasive cancer screening, cancer diagnosis, minimal residual disease (MRD) detection, and monitoring of tumor progression (Diaz and Bardelli 2014; Wan et al. 2017; Corcoran and Chabner 2018; Lustberg et al. 2018; Bronkhorst et al. 2019; Husain et al. 2022).

Levels of single or multiple somatic single-nucleotide variations (SNVs) can be determined through digital droplet polymerase chain reaction (ddPCR) or targeted sequencing (panels) (Diaz and Bardelli 2014; Wan et al. 2017; Corcoran and Chabner 2018; Lustberg et al. 2018; Bronkhorst et al. 2019a; Husain et al. 2022). However, tumor detection from small sample volumes (∼10 mL), such as a single vial of blood, remains challenging due to a combination of factors. For instance, the amount of genetic material is very limited (∼25 ng), corresponding to about 8000 haploid genomes (Alborelli et al. 2019; Chen et al. 2021). At the same time, the genetic material derived from tumor cells is only a small fraction of this, resulting in extremely low levels of mutated alleles at the targeted positions. Consequently, sequencing artifacts and real mutations may be observed at similar frequencies (Bettegowda et al. 2014; Phallen et al. 2017; Cristiano et al. 2019; Zviran et al. 2020). Finally, subclonal expansions of hematopoietic cells are known to contribute to false positive detection of tumor driver mutations and the presence of cancer (Hu et al. 2018; Razavi et al. 2019).

It has been proposed that genome-wide sequencing of cfDNA is a way to circumvent these limitations (Zviran et al. 2020). By looking at the mutations through genome-wide “breadth”, the limited number of genome equivalents becomes less restrictive, enabling the identification of tumor fractions (TFs) ∼100 times lower compared to targeted “depth” in SNV analysis (Zviran et al. 2020). Genome-wide cfDNA can also reveal tumor-specific copy number alterations (CNAs), which occur in more than 90% of solid tumors (Hieronymus et al. 2018; Hoadley et al. 2018; Steele et al. 2022; Martínez-Jiménez et al. 2023). In addition to traditional genomic mutation features, genome-wide cfDNA sequencing also enables the detection of so-called fragmentomics features, such as fragment length and end-motifs (van der Pol and Mouliere 2019; Lo et al. 2021; Liu 2022). Simultaneously evaluating mutational and fragmentomic features in genome-wide cfDNA through a multimodal approach maximizes the capacity to detect ctDNA (Peneder et al. 2021; Moldovan et al. 2024).

A number of sequencing platforms are used for genome-wide cfDNA sequencing, including Illumina sequencing, Oxford Nanopore Technologies (nanopore) sequencing, and PacBio sequencing (Cristiano et al. 2019; Choy et al. 2022; Lau et al. 2023), with Illumina sequencing being the predominant platform owing to its low-cost per base sequenced. However, both Illumina and PacBio sequencing typically require substantial initial investments, which may be prohibitive for smaller hospitals and clinical centers in large parts of the world. Moreover, cost-effectiveness is only achieved if samples are processed in large batches, resulting in prolonged turn-around times as long as several weeks, which is often incompatible with clinical timelines. In contrast, nanopore sequencing offers low-cost, portable, and nonbatched sequencing and is, therefore, increasingly used for cfDNA sequencing (Marcozzi et al. 2021; Lau et al. 2023; van der Pol et al. 2023). The main challenge for implementing nanopore sequencing for the purpose of ctDNA sequencing is the relatively high single-nucleotide error rate (Marcozzi et al. 2021). Multiple strategies were proposed to lower the error rate through repetitive sequencing of the same original molecules (Acevedo et al. 2014; Li et al. 2016; Volden et al. 2018; Wilson et al. 2019; Marcozzi et al. 2021; Thirunavukarasu et al. 2021; Deng et al. 2024). While SNVs are clearly an important asset in ctDNA analysis, no approach has been successfully implemented to sequence genome-wide SNVs in cfDNA using nanopores.

Here, we present genome-wide Nanopore Rolling Circle Amplification (RCA)-enhanced Consensus Sequencing (NanoRCS), a high-accuracy, PCR-free, genome-wide cfDNA sequencing method based on a combination of RCA, and consensus calling of long-read nanopore sequences. The concatemeric RCA products ensure the physical linkage of copies of the same original template, allowing for single-molecule resolution. In the current study, we aim to identify whether NanoRCS allows the detection of tumor-specific SNVs and CNAs, as well as fragmentomic features in cfDNA. This proof of concept study spans three cancer types: common CNA-driven esophageal adenocarcinomas (EACs), rare SNV-driven granulosa cell tumors, and ovarian carcinoma (OVCA) with different subtypes (The Cancer Genome Atlas Research Network 2011, 2017; Nones et al. 2014; Frankell et al. 2019; Roze et al. 2020; Smith et al. 2023).

## Results

### Multimodal genome-wide cfDNA sequencing with NanoRCS

**Figure 1.**
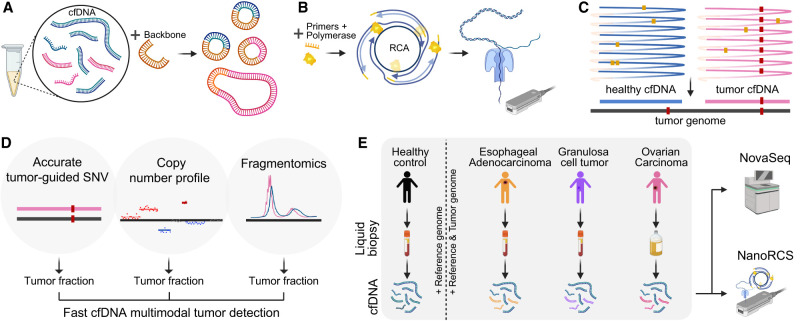
NanoRCS can identify SNVs, CNVs, and fragment size in cfDNA. (*A*) Healthy cfDNA (blue) and tumor ctDNA (pink) are circulated with a high curvature DNA backbone (orange) and form a double-strand circular DNA product. (*B*) DNA primers of random sequence and phi29 polymerase are added to these DNA circles. RCA of double-strand circular DNA templates subsequently creates long concatemers of cfDNA and backbone that are sequenced on a nanopore device. (*C*) Obtained DNA sequences are aligned to the reference genome and undergo consensus calling, resulting in cfDNA consensus sequence with reduced random errors (yellow dots) and retained true variants (red dots). After consensus calling, the tumor mutations can be found in the tumor cfDNA and not in the healthy cfDNA. (*D*) NanoRCS allows simultaneous assessment of fragmentomics, copy number profile, and accurate tumor-guided SNVs. ctDNA fraction can be derived based on all three modalities. (*E*) cfDNA from plasma of healthy controls, EAC and granulosa cell tumor (GCT) patients, and cfDNA from ascites of ovarian carcinoma (OVCA) patients is subjected to genome-wide NanoRCS. We also sequenced the same patient samples with Illumina NovaSeq. Created with BioRender (https://www.biorender.com).

We designed NanoRCS to improve the accuracy of nanopore cfDNA sequencing. First, 5 ng of cfDNA molecules are circularized with a specifically designed flexible DNA backbone (Fig. 1A). The circularized DNA molecules serve as a template to create long concatemers by RCA, which are subsequently sequenced on a nanopore device (Fig. 1B). A high-quality consensus sequence of the cfDNA is finally generated from the concatemers using a consensus algorithm to reduce the sequencing errors (Fig. 1C). A true mutation will be present in all repeats, whereas sporadic sequencing artifacts are reduced by the consensus of multiple (≥3) repeats. NanoRCS offers a precise, multimodal nanopore sequencing-based strategy for cfDNA sequencing through the accurate identification of tumor-informed SNVs along with CNAs and fragmentation length patterns in cfDNA (Fig. 1D).

We performed NanoRCS on cfDNA from the plasma of seven healthy controls (HCs), five patients with metastatic EAC, and two patients with recurrent adult-type GCT of the ovary (including a time-series of one GCT patient), and on cfDNA from ascites of seven patients with OVCA (Fig. 1E; Supplemental Table S1). NanoRCS libraries of cfDNA were sequenced on nanopore MinION and/or PromethION systems. To allow comparison with Illumina sequencing, shallow whole-genome sequencing (WGS) with NovaSeq was performed in parallel on all tumor cfDNA samples and three of the HC samples for which enough cfDNA was available.

A median of 1,570,181 (range 526,943–4,860,826) and 16,620,361 (range 1,403,031–26,816,916) raw reads were obtained on MinION and PromethION, respectively. Reads with ≥3 repeats were used for consensus calling. After deduplication, 51% (range 6%–71%) of raw reads contributed to unique consensus reads on MinION, resulting in a median of 869,287 (range 114,105–1,291,239) unique consensus reads. On PromethION, 35% (range 5%–71%) of raw reads contributed to 4,865,792 (range 896,261–8,905,143) unique consensus reads. Each consensus read contained a median of six subreads (Supplemental Table S1; Supplemental Fig. S1D,E). Library complexity calculations indicated that further sequencing could have yielded additional useful data for the majority of the samples sequenced on MinION (Supplemental Fig. S1B,C; Supplemental Methods), which can be achieved by sequencing on PromethION, or running multiple MinION flow-cells in parallel.

### Low SNV error rate with NanoRCS allows SNV-based tumor detection

**Figure 2.**
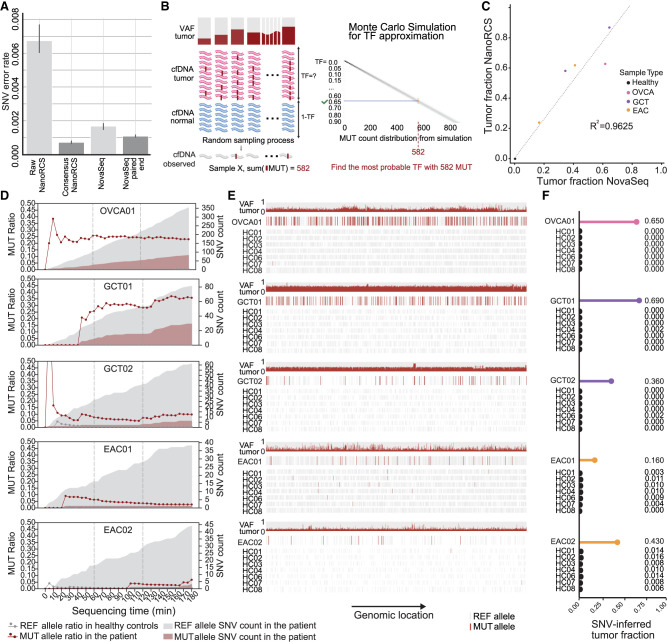
NanoRCS enables SNV detection with a low error rate on Nanopore. (*A*) Single-nucleotide error rate in cfDNA of three HCs using different sequencing methods (*lower* is better). Error bars represent standard deviations. (*B*) Monte Carlo simulations are used to search for the TF that best explains the observations in cfDNA given the known tumor variants and their variant allele frequency (VAF) in the tumor. Created with BioRender (for detailed methods, please see Methods: “Tumor fraction estimation from somatic SNV detection”). (*C*) Correlation of SNV-derived TF between NanoRCS and NovaSeq. (*D*) Real-time assessment of the ratio of the mutant (MUT; red shading) and reference (REF; gray shading) allele at somatic SNV positions during the first 3 h of sequencing in the five liquid biopsy samples with known somatic SNV profile from the tumor biopsy. Red data points and lines indicate the MUT SNV ratios in cancer patients, and dark gray data points and lines in HCs (at level ∼0.00). (*E*) SNV observations in the five liquid biopsy samples with known tumor somatic SNV profile. For each panel, the *top* row shows the VAF of detected mutations in the tumor biopsy, the second row represents the MUT or REF allele observations in the liquid biopsy of the corresponding patient, and the *bottom* three rows represent the observations in three HCs randomly downsampled to the same amount of observation as in the tumor sample. (*F*) Lollipop plots show each sample's inferred TF according to corresponding tumor-informed variants in *C*.

The main shortcoming of native nanopore-based cfDNA sequencing is the difficulty to accurately detect SNVs. We evaluated if NanoRCS lowered the SNV error rate sufficiently to allow accurate somatic SNV detection in cfDNA. In reads obtained from the HC samples that do not overlap with any germline variants, the error rate of non-consensus-called nanopore reads was 0.00674 (Q21) (Fig. 2A). The consensus-calling approach of NanoRCS lowered this error rate ∼9.4 times to 0.00072 (Q31) (Fig. 2A). This error rate was lower than the error rate of Illumina NovaSeq after error correction in overlapping regions of paired-end reads (0.00108; Q30) (Fig. 2A). Notably, the NanoRCS error profile was very uniform across the genome, whereas the GC-rich Chromosome 19 was clearly enriched for sequencing errors in NovaSeq (Supplemental Fig. S2A; Harris et al. 2020).

Next, we performed tumor-informed somatic SNV detection in five cfDNA samples where tumor biopsy sequencing is available (Supplemental Table S2). Tumor-informed somatic SNV detection in cfDNA samples involves counting MUT alleles versus wild-type alleles in the cfDNA molecules that overlap with mutations observed in the tumor biopsy. The mutation fraction was subsequently calculated and compared between each tumor cfDNA sample and HC background levels. In total, between 10 and 582 somatic SNVs were detected in each of the five tumor samples (Fig. 2E; Supplemental Fig. S2C–L). False positive SNV calls in HC samples were very low for OVCA01, GCT01, and GCT02, at only 0–3 observations per sample, while in EAC samples a higher background of between 0 and 18 false positive SNV calls was observed (Fig. 2E; Supplemental Fig. S2C–L). In EAC samples, somatic SNVs were determined by sequencing formalin-fixed paraffin-embedded (FFPE) tumor biopsies, which is known to cause false positive somatic SNV calls (Do and Dobrovic 2015; Robbe et al. 2018). This implies that the majority of the false positive calls in EAC cfDNA may have been, in fact, false positive calls in the tumor biopsies instead of cfDNA. Regardless, MUT alleles were significantly higher in all patients versus HCs (Fisher's exact test, *P*-value < 1.1 × 10^−11^), confirming that NanoRCS can capture tumor-informed somatic SNVs in cfDNA confidently.

### Real-time cfDNA sequencing and SNV-based tumor fraction inferences

Nanopore sequencing uniquely offers real-time sequencing, where results can be analyzed while sequencing. To demonstrate how this can be leveraged, we determined the minimum sequencing time required for finding MUT alleles in tumor samples. Within 20–110 min of sequencing, MUT read counts exceeded the HC background levels in all five samples (Fig. 2D). For samples with TFs lower than 0.1, however, simulations showed that a longer sequencing time may be required (Supplemental Fig. S3; Supplemental Methods).

To reliably estimate the TF from SNV data, we developed a Monte Carlo simulation approach. The simulations incorporate the observed number of tumor-informed SNVs, the VAF of each SNV in the tumor, and the sequencing error rate to estimate the most probable TF (Fig. 2B; Methods: “Tumor fraction estimation from somatic SNV detection”). Using this approach, we found that TFs of the patient samples were between 0.16 and 0.69 (Fig. 2F; Supplemental Table S3), which was more than 40× higher than HCs (∼0.004) (Fig. 2F). The SNV-based TFs observed through NanoRCS and NovaSeq were highly correlated (*R*^2^ = 0.96) (Fig. 2C), confirming that the SNV error rate is sufficient to estimate ctDNA fraction in liquid biopsies reliably.

### CNA-based tumor detection using NanoRCS

**Figure 3.**
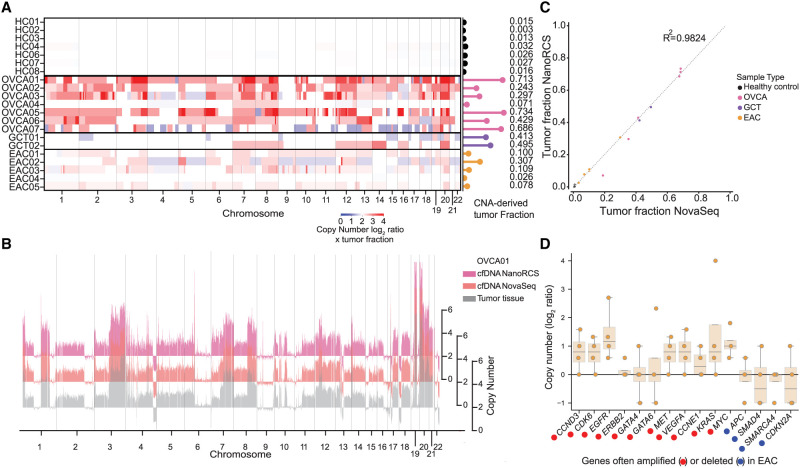
NanoRCS identifies CNA in cfDNA of tumor samples. (*A*) CNAs and derived TF (lollipops) for cfDNA samples across different sample types. (HC) healthy controls, (OVCA) ovarian carcinoma, (GCT) granulosa cell tumor, (EAC) esophageal adenocarcinoma. Tumor copy numbers were calculated by multiplying the copy number in the liquid biopsy by the TF in that liquid biopsy. Red indicates copy number gain and blue indicates copy number loss. Color intensity indicates the CNA multiplied by the TF in cfDNA. (*B*) The CNA profile, obtained using NanoRCS on cfDNA (pink), NovaSeq on cfDNA (orange), and from tumor tissue (gray), is concordant in patient OVCA01. The *x*-axis indicates the genomic position on the chromosomes (1 Mb bins), and the *y*-axis indicates copy numbers. (*C*) Correlation of CNA-derived TF between NanoRCS and NovaSeq. (*D*) NanoRCS copy number of genes often amplified (red) and deleted (blue) according to literature. The boxplots show the observed copy number distribution across the four EAC samples in this study. Each data point represents a single (EAC) sample.

CNAs represent another important feature of the tumor that is reflected in the ctDNA. Using ichorCNA, a tool optimized for CNA analysis on ultra-low-pass genome-wide sequencing data (Adalsteinsson et al. 2017), CNAs were observed in 13 out of 14 patient cfDNA samples (Fig. 3A,B; Supplemental Fig. S4A). Five out of seven OVCA ascites samples showed numerous copy number gains indicative of whole-genome amplifications, which fits with the high-grade serous OVCA subtype of most of these samples (Fig. 3A; Supplemental Table S2; Bielski et al. 2018; Cheng et al. 2022; Yang et al. 2022). EAC samples exhibited numerous copy number gains and losses (Fig. 3A), in line with the fact that this cancer is typically driven by CNAs (Nones et al. 2014; The Cancer Genome Atlas Research Network 2017; Frankell et al. 2019), while SNV-driven GCT (Roze et al. 2020) patients displayed fewer CNA regions (Fig. 3A). In sample EAC04, we did not observe a clear CNA profile in the cfDNA. This could be due to a low TF or absence of CNAs in this sample. CNA profiles from NanoRCS and NovaSeq cfDNA (Supplemental Fig. S4A) were highly correlated for most samples (Pearson's correlation = 0.715) (Supplemental Fig. S4B), confirming that NanoRCS can capture CNA profiles in cfDNA reliably.

The cfDNA-derived CNA profiles were compared to the CNA profiles obtained from the tumor biopsy samples, if available. For four out of five samples, CNA patterns were similar (Fig. 3B; Supplemental Fig. S4C–F). The more dissimilar sample, GCT02, showed additional CNAs in cfDNA compared to the sequenced tumor tissue biopsy (Supplemental Fig. S4D), suggesting ongoing tumor evolution. CNA profiles obtained through NanoRCS were concordant with those obtained with NovaSeq sequencing for this patient.

For samples with at least one CNA, ichorCNA allows direct estimation of the TF, with inferred TFs above 0.03 considered to be reliable (Adalsteinsson et al. 2017). In 13/14 patient samples, we reliably detected TFs from the cfDNA-derived CNA profile. While HCs had a median TF of 0.016 (range 0.003–0.032), TFs of cancer samples ranged between 0.026 and 0.73 (median = 0.31), with OVCA ascites samples generally displaying high TFs (median = 0.43) compared to plasma samples (median: 0.10) (Fig. 3A; Supplemental Table S3). The CNA-derived TF of EAC04 was too low (0.026) to be detected reliably with NanoRCS. However, we detect a TF of 0.034 in the same sample using NovaSeq, suggesting that this sample does have a detectable TF (Fig. 3A; Supplemental Fig. S3A). The CNA-based TFs observed through NanoRCS and NovaSeq were highly correlated (*R*^2^ = 0.98) (Fig. 3C), confirming that NanoRCS combined with ichorCNA can correctly identify TFs through CNA analysis.

To examine if the CNAs in cfDNA samples corresponded to known driver CNAs, the copy number of frequently amplified and deleted genes in tumors was determined in the cfDNA (Fig. 3D; Supplemental Fig. S5). All patients with EAC in this study have *ERBB2*-negative tumors, matching with the observation that *ERBB2* was copy number neutral (Supplemental Table S2). In the other 10 commonly amplified and four commonly deleted genes in EAC, concordant CNAs were observed in 7/10 and 2/4 genes, respectively (Fig. 3D). We performed similar analyses for CNA driver genes in OVCA and GCT and found that 12/15 and 5/8 of the genes, respectively, that are frequently hit by copy number changes in these solid tumors showed similar patterns in the cfDNA data (Supplemental Fig. S5). The observation of gain and losses of genes through cfDNA could be useful for detecting driver CNA events, especially in EAC where there are often no SNV-drivers.

### Fragmentomics-based tumor detection using NanoRCS

**Figure 4.**
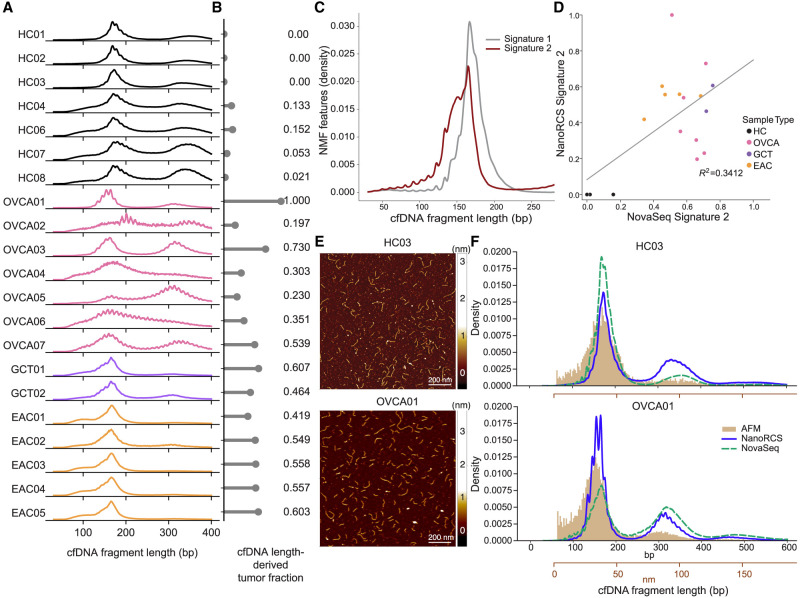
NanoRCS captures cfDNA fragmentation length distribution indicating tumor presence. (*A*) cfDNA fragmentation length profiles for each sample, categorized by sample type. (HC) healthy controls (black), (OVCA) ovarian carcinoma (pink), (GCT) granulosa cell tumor (purple), (EAC) esophageal adenocarcinoma (orange). The profiles display the distribution of cfDNA fragment sizes ranging from 30 to 400 bp. (*B*) TF for each sample derived by mapping the length profile to *Signature 2* of the nonnegative matrix factorization (NMF) on a reference set (see Methods). TF is annotated to the *right* of each bar, ranging from 0.14 to 1.0 for tumor samples. (*C*) Representative NMF cfDNA length profiles adapted from Renaud et al. (2022). for two signatures: Signature 1, predominantly observed in healthy individuals, and Signature 2, indicative of tumor-derived cfDNA. (*D*) Scatter plot correlating the fragmentomics NMF-derived TF of NanoRCS and NovaSeq against each other (*R*^2^ = 0.3412). (*E*) Atomic force microscopy (AFM) images visualizing HC03 (*top*) and OVCA01 (*bottom*) cfDNA fragments (yellow). Scale bar, 200 nm. (*F*) Density plots comparing the length distribution obtained from three different techniques (blue, NanoRCS; green, NovaSeq; yellow, AFM) in sample HC03 (*top*) and OVCA01 (*bottom*). Conversion of the *x*-axis in nm (brown) to the *x*-axis in bp (black) is based on the calculation of the DNA ladder where *L*_bp_ = (*L*_nm_ + 10)/0.341. AFM imaging (brown) appears to better represent the shorter fragments, while both sequencing methods enriched for longer fragments, especially NanoRCS (blue).

cfDNA has a fragment length mode at ∼167 bp and at ∼332 bp, corresponding to single or di-nucleosomal DNA (Bronkhorst et al. 2019; Yu et al. 2021), while ctDNA fragments are slightly shorter and exhibit a prominent ∼10 bp periodicity (Mouliere et al. 2018; Udomruk et al. 2021). In line with this, NanoRCS-derived fragmentation patterns for the GCT and EAC plasma samples consistently showed ∼4–5 bp shortening of the first peak (Fig. 4A; Supplemental Table S4). The OVCA ascites samples displayed variable patterns, typically with a distinct 10 bp periodicity (Fig. 4A). This might reflect differences in release mechanisms or removal of cfDNA specific to the abdominal environment compared to the bloodstream. Notably, previous work (Werner et al. 2021) found more concordant fragmentation patterns between ascites and blood, indicating that further investigation into the relation between the biofluid and fragmentation patterns is required.

To estimate TF from fragmentation patterns, we normalized the cfDNA length distribution within the 30–220 bp range and fitted it to a two-component NMF model derived previously (Fig. 4B–D; Lee and Seung 1999; Renaud et al. 2022). The contribution of Signature 2 served as an estimator for TF (Fig. 4B,C; Supplemental Figs. S6, S7). The seven HC samples showed a fragment length-derived TF of 0–0.152, whereas the patient samples exhibited contributions ranging from 0.197 to 1.00 (Fig. 4B; Supplemental Table 2). The correlation of NanoRCS-derived and NovaSeq-derived TF was modest (*R*^2^ = 0.34) (Fig. 4D). Taken together, this indicates that, while the fragmentomics-based TF measure may not be very precise, the fragmentomics-based TF measure can distinguish tumor samples from HCs.

To verify if the length differences between HC and tumor samples found in NanoRCS reflect true differences or bias from the sequencing, we employed an orthogonal measurement, AFM imaging, which measures DNA length with ∼3 bp resolution through direct visualization of DNA on a flat surface (Binnig et al. 1986; Mouliere et al. 2014). Between 19,274–107,424 molecules per condition were computationally analyzed (Fig. 4E,F; Supplemental Fig. S8). Consistent with the sequencing results, AFM detects shorter DNA fragment lengths for the tumor samples compared to the HCs (Fig. 4; Supplemental Fig. 8K,L). Comparison of AFM imaging derived cfDNA length profiles to the length profiles obtained by NanoRCS and NovaSeq suggested that sequencing methods enrich for fragment lengths above 200 bp, while the AFM method was better at detecting shorter fragments <150 bp (Fig. 4F). NanoRCS was able to capture the second (∼330 bp) and third (∼500 bp) peak in the fragment length distribution more clearly than NovaSeq and AFM. This analysis reveals that while the precise measurements of peak lengths in cfDNA and the proportion of longer to shorter sequences differ based on the method applied, there is a consistent trend of shorter cfDNA fragments in cancer samples as opposed to healthy ones (Fig. 4F).

### Detecting low tumor fractions using multimodal NanoRCS

**Figure 5.**
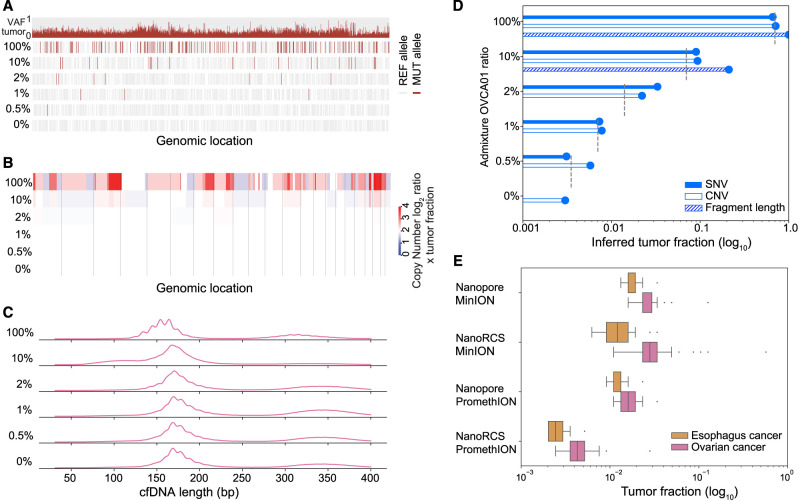
MRD detection using NanoRCS. (*A*–*D*) Admixture experiment with different percentages of OVCA01 versus HC02 cfDNA (100%, 10%, 2%, 1%, and 0.5%, 0% mixtures). (*A*) SNV observations for all dilutions. The *top* row shows the VAF of detected mutations in the tumor biopsy and the following five rows represent the MUT or REF allele observations in the different admixtures. (*B*) CNAs for all dilutions. Red indicates copy number gain and blue indicates copy number loss. Color intensity indicates the CNA multiplied by the TF in cfDNA. (*C*) cfDNA fragmentation length profiles for all dilutions. The profiles display the distribution of cfDNA fragment sizes ranging from 30 to 700 bp. (*D*) Inferred TF of each of the three NanoRCS modalities is shown in log-scaled bars. SNV (solid), CNA (nofill), and fragmentation length (striped) are compared to theoretical expected TFs (gray dashed lines). (*E*) Simulation results to assess the lowest TFs detectable for different platform throughputs (MinION vs. PromethION) and with or without consensus calling for Esophagus and OVCA. Simulation characteristics were obtained from PCAWG (see Methods).

By combining multimodal cfDNA signals, NanoRCS detects disease in all tumor samples and not in healthy samples (Supplemental Fig. S9; Supplemental Table S2). To determine the limit of detection, admixture ratios of OVCA01 and HC02 (0.5%–10% OVCA01; corresponding to TFs of 0.004–0.071) (Fig. 5A–D) were sequenced using NanoRCS. TF estimates from fragment length and CNA indicated that TFs down to 0.071 could be detected (Fig. 5B–D), with the CNA profile at TF = 0.071 still consistent with the original OVCA01 sample. However, at TFs <0.03, the ichorCNA profile started to deviate from the CNA profile observed in sample OVCA01, confirming that these CNAs at low TFs were unreliable (Fig. 5B,D). Notably, through SNV analysis, the presence of ctDNA was confirmed in all admixtures, including the lowest TF of 0.004 (>80-fold above the HC background levels). The admixture experiment suggests that genome-wide tumor-informed SNV analysis allows the detection of the lowest TFs, which is required for proper MRD detection (Fig. 5A,D).

To further deduce the lowest limit of detection, 300 million in silico Nanopore and NanoRCS MinION and PromethION cfDNA sequencing runs were simulated for 50 EAC and 100 OVCA patients while taking into account a realistic sequencing throughput and error rate (Supplemental Fig. S10). We then identified the lowest TF detectable for each simulated patient. Comparing PromethION to MinION and raw to consensus-called NanoRCS clearly showed that both a lower error rate and a higher throughput could further improve MRD detection (Fig. 5E; Supplemental Fig. S10D). We also observed that higher VAFs and higher variant counts in samples contributed to improved detection of lower TFs within a cancer type (Supplemental Fig. S10). More than 95% of the samples could be distinguished above background at a TF of 0.0024 (range 0.0020–0.0052) in esophagus cancer samples and TFs of 0.0043 (range 0.0024–0.0281) in OVCA samples (Fig. 5E).

**Figure 6.**
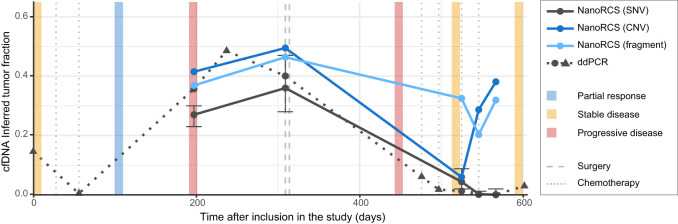
NanoRCS TFs of patient GCT02 during treatment. ctDNA fraction estimations in patient GCT02 were obtained using ddPCR (dotted line) and NanoRCS (solid lines); the three colors of solid lines represent the three modalities. A time period of 600 days after inclusion in the study is displayed. Circles represent samples that were analyzed using both ddPCR and NanoRCS, whereas triangles represent samples that were only assessed using ddPCR. Clinical events such as disease state (partial response, stable disease, progressive disease) are shown by colored blocks and interventions (surgery, chemotherapy) by vertical lines. Error bars for the SNV modality represent 95% confidence intervals.

To further demonstrate the utility of NanoRCS in MRD, we retrospectively applied NanoRCS to cfDNA sampled at five time points of patient GCT02 (Fig. 6; Supplemental Table S2). GCT02 was diagnosed with GCT 17 years before WGS of the tumor tissue, and the disease became progressive with unresectable metastases 11 years later. Around the time of cfDNA time-series measurements (indicated as day 0–602), the patient had multiple chemotherapy treatments and surgeries, which led to a temporary stable disease (days 518, 595). The patient, however, relapsed shortly after and died on day 760. For this patient, ddPCR-based assessments of *FOXL2* mutation were performed at 11 time points (Groeneweg et al. 2021). Using NanoRCS, TF estimates obtained from all three modalities closely matched the TF values derived from ddPCR at the initial time points (days 237, 309). At later time points (days 525, 546, 567) when the patient had stable disease; however, the TF became very low with SNV measurements (both ddPCR and NanoRCS), while the presence of tumor-derived cfDNA increased drastically and became evidently high with CNA and fragmentomics analysis. cfDNA-based CNA analysis revealed the emergence of a distinct CNA profile with different gains and losses after day 497, suggesting that a new subclone became predominant (Supplemental Fig. S11). The increased CNA and fragmentomics inferred TFs at late time points corresponded to the patient disease progression at day 720 and death at day 760. This experiment demonstrates that simultaneous evaluation of SNV, CNA, and fragmentomics features in genome-wide NanoRCS enables the most efficient detection of cancer in cfDNA.

## Discussion

We introduce NanoRCS, a rapid, highly accurate, nanopore-based sequencing technology capable of attaining genome-wide cfDNA profiles in a single sequencing run. As shown previously, genome-wide tumor-informed SNV detection in cfDNA can alleviate the limitations associated with detecting one or a few mutation targets in sample volumes with a limited number of genome equivalents present (Zviran et al. 2020). In addition to SNVs, NanoRCS can simultaneously analyze CNAs and fragmentation patterns in cfDNA. We also show that the technique is compatible with different biofluids. As a result, NanoRCS offers a comprehensive and accurate representation of all subclones present in a tumor, improving the ability to detect tumor presence, monitor tumor progression, and identify treatment resistance.

The complementarity of cfDNA modalities is exemplified by our patient time-series analysis and dilution experiments. The TF became (undetectably) low through SNV analyses, while the CNA and fragmentomics modalities provided clear and early evidence for the emergence of a new tumor clone. A distinct CNA profile in late time points suggests ongoing tumor evolution, which may go unnoticed when considering single-driver events through ddPCR. On the other hand, the admixture experiments showed that only SNV analysis is suitable for detecting TFs below 3% and the SNV modality was most consistent, whereas there was more variability in CNA and fragmentomics analyses. As both tumor evolution and multiclonality are known to occur frequently in tumors (Roerink et al. 2018), we envision that capturing multimodal signals in cfDNA will provide the most complete picture.

Lowering the sequencing error rate is especially crucial for cfDNA sequencing because real mutations can occur at a similar frequency as sequencing artifacts, making true variants impossible to detect. There is a continued effort by multiple sequencing vendors to lower the single-molecule error rate to Q40 and beyond. Nevertheless, for uninformed SNV analysis in liquid biopsies, a Q70 or higher will be required to detect TFs > 1% in a cfDNA sample with cancer (Extended Methods). NanoRCS improved the error rate of Nanopore-based sequencing to the equivalent of Illumina NovaSeq with paired-end error correction, PacBio HiFi reads, and Avidity sequencing (Hon et al. 2020; Stoler and Nekrutenko 2021; Arslan et al. 2024). In addition to NanoRCS, other avenues to lowering sequencing errors are being explored for various platforms. Most notably, amplification with unique read identifiers (UMIs) has been demonstrated for both NovaSeq and Nanopore (Kivioja et al. 2012; Karst et al. 2021) as a potent way of producing very high single-molecule accuracies. However, UMI-based methods suffer from clustered amplification errors (Lou et al. 2013). In contrast, NanoRCS is PCR-free and relies on RCA where consensus calls are made from connected copies of the original template, preventing amplification of errors introduced by PCR.

Notwithstanding the importance of lowering the sequencing error rate, our simulations on the efficacy of the SNV modality for tumor presence detection demonstrate that there is a clear trade-off between coverage and error rate. We conclude that not only low error rate is essential, but a reasonable throughput (e.g., 0.71× at Q31) is also required to capture sufficient mutated bases in low TF samples. This is particularly important for patients with early stage cancer and for samples and tumor types with low tumor mutational burden. In theory, NanoRCS lowers throughput capacity by sequencing the same molecule in a single RCA product multiple times. However, a recent report of sequencing native cfDNA with MinION of which 5 ng of input cfDNA resulted in only 3 million reads (∼0.15–0.20×) after demultiplexing (Lau et al. 2023), suggesting that the numbers of unique read NanoRCS provides are not lower than native cfDNA sequencing on Nanopore.

An exciting aspect of the nanopore platform is to deliver real-time sequencing results (Vermeulen et al. 2023; Weilguny et al. 2023). Indeed, it is possible to detect ctDNA for high TF samples based on only 20 min of sequencing on a PromethION system using NanoRCS. Moreover, the nanopore platform has experienced continuous improvements in recent years, majorly increased throughput, and substantially reduced error rates (Delahaye and Nicolas 2021). These improvements are likely to continue in the coming years, which also directly benefits the throughput and error rates of NanoRCS. It should be noted that, although NanoRCS enables more accurate SNV detection in cfDNA, native Nanopore sequencing has the advantage of incorporating methylation as an additional modality that can be captured (Simpson et al. 2017; Lau et al. 2023). Future improvements of NanoRCS include encompassing even more modalities, such as additional fragmentomic features, to further increase comprehensiveness.

NanoRCS requires low setup costs, thereby lending itself for quick introduction into the clinic and adoption in resource-limited parts of the world. To successfully bring NanoRCS into clinical practice, large multicenter clinical studies with patients with various cancer types across different tumor stages are required. These studies are crucial for validating the efficacy and reliability of NanoRCS across diverse oncological contexts such as early diagnosis, monitoring treatment response, and detecting MRD. In addition to robust clinical validation, securing CE marking and obtaining FDA, and in vitro diagnostic (IVD) medical devices regulatory approvals will ensure that NanoRCS meets international standards for safety and efficacy. Within Europe, CLIA-certified laboratories and clinics also have the option to use research-only kits for clinical purposes after completing a rigorous internal validation process, bridging the gap between research and practical application. This regulatory flexibility allows for the accelerated introduction of cutting-edge technologies like NanoRCS into routine clinical workflows, ultimately enabling patients to benefit from this innovative technology much sooner.

## Methods

### Tumor tissue sample collection and cellular DNA sequencing

Tumor DNA of EAC samples was extracted from FFPE slides of tumor tissue. Data from WGS of tumor DNA extracted from fresh frozen tissue in GCT and OVCA samples were obtained from previously published studies of matched patients (Kopper et al. 2019; de Witte et al. 2020). The use of these human specimens for research purposes was approved by the Medical Ethics Committee of the UMC Utrecht (14–472 HUB-OVI, 17–868, and 20/055) and by the Medical Ethics Committee of the Amsterdam UMC (METC 2013_241), respectively. All participants provided written informed consent. A detailed description of sample collection and WGS can be found in Supplemental Methods.

### Somatic variant analysis in cellular DNA sequencing of tumor tissue

WGS data of tumor biopsies and matched germline samples was mapped to the human reference genome (hs37d5). The WGS data underwent preprocessing steps using the Sarek (Hanssen et al. 2024) nf-core (Ewels et al. 2020) NextFlow pipeline (nf-core/sarek v3.1.2), adhering to the recommended GATK (Van der Auwera and O'Connor 2020) best practices. Default options were activated, including: adapter trimming (fastp 0.23.2) (Chen 2023), alignment (BWA-MEM, v0.7.17-r1188), MarkDuplicates (GATK4, v4.3.0.0), and Base quality score recalibration (GATK4, v4.3.0.0). Variant calling was performed subsequently by Strelka (v2.9.10) (Saunders et al. 2012) and Mutect2 (GATK4, v4.3.0.0) by providing paired tumor and normal samples. Single-base somatic variants on autosomal chromosomes with a Strelka filter “PASS” that intersect with Mutect2 results are selected. Subsequently, variants with filter labels: “germline” and “panel of normal” were removed to remove potential false positive calls. For EAC samples, an extra step of selecting “PASS” variants in Mutect2 was performed to reduce the amount of false positive calls. With these filtering criteria, a list of tumor-informed somatic SNV was recorded as a VCF file. These VCFs were used in the following Methods section: “Tumor-informed SNVs detection in NanoRCS”.

### cfDNA isolation from plasma and ascites

Plasma and ascites were collected and stored fresh frozen in −80°C for cfDNA extraction (see Supplemental Methods for detailed information). cfDNA was isolated from 5 mL plasma or 5 mL ascites using the Quick-cfDNA Serum & Plasma Kit centrifugation protocol (Zymo Research) for HC, OVCA, GCT samples, and QIAamp Circulating Nucleic Acid Kit (Qiagen) for EAC samples, and then stored in Milli-Q at −80°C. DNA concentration was determined by the High Sensitivity Assay Kit (Thermo Fisher Scientific) using a Qubit fluorometer. Between 5 and 25 ng of cfDNA was obtained from the human specimens. These cfDNA are used for downstream NanoRCS and Illumina NovaSeq sequencing.

### NanoRCS library preparation of cfDNA

NanoRCS is developed in order to generate RCA-derived concatemers of linear and circular cfDNA for subsequent nanopore sequencing. While circular cfDNA can serve as an RCA substrate as it is, the linear cfDNA needs to be circularized. The circularization reaction used in this study is mediated by a proprietary DNA linker called backbone. Several variations of the NanoRCS were used during this study. Different circularization conditions were evaluated. In particular, we have tested the effect of (1) different amounts of cfDNA input, (2) different backbone sequences, (3) de-phosphorylation of the cfDNA before the circularization reaction, (4) circularization using backbones and cfDNA with blunt- or tailed-ends, and (5) postcircularization digestion of backbone–backbone concatemers. Each method eventually yielded similar concatemeric DNA as a product, and we did not find any noticeable difference in the consensus reads generated using the different protocols. Detailed steps and variations of the protocols can be found in the Supplemental Methods.

### NanoRCS sequencing and consensus base calling

Prepared sequencing runs are submitted for standard nanopore sequencing with MinION or PromethION for 72 h. Sequencing is done via the MinKNOW interface using the SUP base caller model. Multiple FASTQ files are derived from each nanopore run. Data are then processed via the CyclomicsSeq bioinformatics pipeline automated with NextFlow workflow language to generate consensus sequences. In short, nanopore reads are aligned to the human reference genome (hg37d5) with minimap2 (Li 2018). A consensus algorithm takes aligned BAM reads and constructs consensus alignment sequences based on a majority vote for each position, per read. Consensus base quality is calculated by aggregating the quality score for all reads contributing to the final consensus. The remaining backbone sequences are subsequently trimmed from both 3′ and 5′ ends. Reads mapped to the same genomic coordinates with ±1 bp are merged. We opted for hg37d5 in our analyses, as ichorCNA is most optimal for this genome version. Nevertheless, NanoRCS is also compatible with newer genome versions and we do not expect that the results will significantly change with a newer version.

### NovaSeq cfDNA library preparation, sequencing, and base calling

Illumina sequencing libraries are prepared by incorporating 1–10 ng of cfDNA with the ThruPLEX Plasma-seq Kit by Takara Bio, following the guidelines provided by the manufacturer. The quality of these libraries was assessed using the Agilent 4200 TapeStation System, employing the D1000 ScreenTape Analysis assay from Agilent for precise evaluation. Following quality control, the libraries were pooled in equal molar concentrations and sequenced. Two sequencing runs were performed on an Illumina NovaSeq 6000 system, utilizing 150 bp paired-end runs and S4 flow-cells.

Illumina sequencing output is mapped to the human reference genome (hs37d5). Briefly, FASTQ reads are trimmed with BBDuk (v38.79) (Bushnell 2024) before mapping with BWA-MEM (v0.7.17). Duplicates in BAM files are removed with Picard MarkDuplicates (Picard v2.22.2, http://broadinstitute.github.io/picard). BAM files are filtered to remove PCR duplicates, nonprimary alignments, and reads with a mapping quality of less than five.

### cfDNA SNV detection error rate

SNV detection error rate was established by sequencing cfDNA of HCs with known genome references. The germline variants of HCs were identified with haplotypecaller (GATK4, v4.3.0.0). Two different sequencing methods were processed in four manners and the error rate was compared: raw NanoRCS, consensus NanoRCS, Illumina NovaSeq, and Illumina NovaSeq with paired-end correction. For raw NanoRCS, BAM files are obtained by mapping the nanopore FASTQ files obtained with the NanoRCS wet-lab protocol to the human reference genome (hs37d5) with minimap2, settings “-ax map-ont -m 1 -n 10 -s 20” and subsequently keeping the first alignment of each Nanopore FASTQ read. Illumina NovaSeq and NanoRCS alignment BAM files preprocessing steps were as described in the sections above. Subsequently, all alignment BAM files are filtered to include only primary alignment reads with a mapping quality of at least 60, then overlapped with the germline variants of HCs with BEDTools intersect (Quinlan and Hall 2010). Reads overlapping with a germline variant are removed from the analyses. The remaining reads are supposed to be exactly the same as the reference genome for all samples. The edit distance of each read to the reference is defined as sequencing errors. We randomly subsample 400,000 nonoverlapping reads and calculate error rates in all reads. The error rate is calculated by the mismatches excluding indels divided by mapped read length.

### Tumor-informed SNV detection in NanoRCS

VCF files from germline and somatic variant analysis in tumor biopsies with matched germline samples are acquired as described in the Methods section “Somatic variant analysis in cellular DNA sequencing of tumor tissue.” These somatic variants specific to the tumors serve as the markers in tumor-informed SNV detection. All mapped reads in the BAM files that overlap with these SNV positions are recorded. The allele overlapping with tumor-informed SNV is noted, reference allele, MUT allele, or erroneous allele (an alternative allele that was not the MUT allele in tumor VCF. Reads overlapping with these identified variants are annotated with a timestamp of sequencing for NanoRCS. For each time period, the number and ratio of MUT alleles and/versus the reference allele are recorded. The number of MUTs, reference, and erroneous alleles is recorded for TF estimation for NanoRCS and Illumina NovaSeq.

### Tumor fraction estimation from somatic SNV detection

To estimate TF from cfDNA mixtures, we employed a Monte Carlo simulation approach. cfDNA mixtures are composed of fragments from both healthy cells and tumor cells. Because of the probabilistic nature of observing a MUT or REF allele in tumor-derived cfDNA. Each variant position from tumor-derived cfDNA has a probability *P* of being observed (adjusted for tumor purity VAF); for example, if the tumor purity is 60% and the VAF is 50%, then there is a 30% probability of observing that particular MUT allele. Conversely, there is a *1*−*P* probability of observing a REF allele. For healthy-cell-derived cfDNA, the probability of observing a MUT allele is 0. To derive the possible outcome of a given set of MUT alleles and their associated VAFs, we systematically vary the percentage of tumor-derived cfDNA from 0.00 to 1.00 in 100 discrete linear steps. For each percentage, we count the number of observed MUT alleles by randomly sampling each allele based on their probability. We repeat this process for 10,000 trials and collect the observed MUT allele frequencies per TF. The most likely TF in each sample was inferred by identifying where the highest percentage of simulations aligned with the observed distribution of cfDNA sources. The confidence interval is derived from the TFs that fall at the 2.5% and 97.5% of the simulated distribution. If the inferred TF is lower than 0.05, we repeated the same process with 100 discrete log steps ranging from 0.00 to 0.10 to obtain a more fine-grained TF estimation.

### Somatic copy number alteration analysis and tumor fraction inference

We use ichorCNA (Adalsteinsson et al. 2017) software (commit 5bfc03e) to calculate and visualize the copy number alterations and infer copy number derived TF in all samples. Customized panels of normal (PoN) file was created and used for NanoRCS runs (the PoN file is available on the GitHub repository). Two parameter combinations were tested for all samples, one allows flexibility of high ploidy and high copy number; the other is optimized for low TF samples (MRD setting). If the inferred TF was below 0.03, the MRD setting was applied. The optimal solution was chosen from the provided solutions according to the principles the author suggested and the prior knowledge of ploidy in tumor tissue samples. The selected solutions are provided in Supplemental Table S6, and please refer to the Supplemental Methods and Supplemental Table S5 for a detailed description on settings.

### Analyzing CNA on tumor-specific copy number events

Tumor-specific copy number events are tumor-type specific. Specific copy number events were curated from literature for EAC (The Cancer Genome Atlas Research Network 2017; Frankell et al. 2019), OVCA (de Witte et al. 2022), and GCT (Roze et al. 2020). The list of the following genes was curated. For EAC: *KRAS*, *VEGFA*, *EGFR*, *ERBB2*, *GATA4*, *GATA6*, *MYC*, *CDKN2A*, *SMAD4*, *SMARCA4*, *CCND1*, *CCND3*, *CCNE1*, *CDK6*, *MET*, *PTEN*, *APC*, *CCNE1*. For OVCA: *KRAS*, *MYC*, *CCNE1*, *TP53*, *NF1*, *CDKN2A*, *RB1*, *MAP2K4*, *BRCA1*, *BRCA2.* For GCT: *FOXL2*, *TP53*, *TERT*, *DICER1*. Genomic coordinates of genes of interest were retrieved from Ensembl biomart version GRCh37. The copy number of the one megabase bin overlapping with these genes was determined to be the copy number of the gene.

### Nonnegative Matrix Factorization–derived tumor fraction by decomposing cfDNA fragment lengths

NMF is a nonsupervised method where a matrix V is factorized into two nonnegative matrices W and H. The weight matrix W, has as many rows as the input matrix and represents the contributions of each cfDNA source to each sample. The number of cfDNA sources is a hyperparameter that needs to be set in advance. Renaud et al. (2022) used NMF to determine the contribution of different cfDNA sources to fragment length signatures in 86 prostate cancer samples. We adapted the two signatures extracted from this analysis, selected the region between 30 and 220 bp, and used them as signatures to decompose the cfDNA source in our sample sets. The fragments between 30 and 220 bp are selected and normalized to 1. NMF with fixed signatures with function non_negative_factorization from sklearn.decomposition (v1.1.2) is applied to obtain cfDNA source contribution for each sample. All values are capped at 1.0.

### Atomic force microscopy image analysis for cell-free DNA length determination

cfDNA samples and a commercial DNA ladder (Thermo Scientific GeneRuler 50 bp DNA Ladder) were prepared and imaged with a Nanowizard Ultraspeed 2 AFM (Bruker). For detailed sample preparation steps, please check the Supplemental Methods. Data processing used the software SPIP (Image Metrology, v6.5) and involved background correction using global correction with a third order polynomial and line-wise correction of the third degree. The z-offset is set to the mean pixel height after background correction, corresponding to the mean height of the mica surface.

To quantify DNA length distributions from the AFM images, we employed the Particle Analysis pane in the Scanning Probe Image Processor (SPIP). A threshold detection level of 0.6 nm with respect to the z-offset was used, and we included a postprocessing step to eliminate small speckles with a surface-projected area <100 nm^2^. The resulting features were traced by skeletonization and the reported lengths values are the fiber lengths, i.e., the longest segment of a one-pixel wide branched line obtained by thinning the 2D-projected surface area of each chain.

### Determining the lowest TF detectable with the generation of cfDNA admixture

We generated cfDNA admixture in the laboratory with samples OVCA01 and HC02. 10%, 2%, 1%, 0.5%, admixture of OVCA01:HC02 ratio. A 10 times or 100 times diluted stock solution of OVCA01 was used to prepare a more precise admixture. Final solutions of 5 ng DNA cfDNA admixture were subjected to NanoRCS library preparation, followed by sequencing runs with MinION and PromethION flow-cells as described above (and in Supplemental Table S1). Inference of TF in three modalities, including SNV, CNA, and fragmentation length, can be found in previous sections.

### Determining the lowest TF detectable with simulation on PWACG tumor patient samples

Genome-wide somatic SNV profiles of 50 EAC and 100 OVCA patients were obtained from The ICGC/TCGA Pan-Cancer Analysis of Whole Genomes Consortium (The Cancer Genome Atlas Research Network et al. 2013; The ICGC/TCGA Pan-Cancer Analysis of Whole Genomes Consortium 2020) (PCAWG; https://dcc.icgc.org/pcawg). Real mutation numbers and VAFs were used as input for the simulations (Supplemental Fig. S9B,C). We then generated in silico genome-wide sequencing data sets for each patient (Supplemental Fig. S9A) at 51 different TFs (TF of 0 and 50 TFs between 0.001 and 1, logarithmic) and for four techniques: native Nanopore sequencing on MinION (0.25× coverage), native Nanopore sequencing on PromethION (3× coverage), NanoRCS on MinION (0.04× coverage), and NanoRCS on PromethION (0.8× coverage). Error rates for each of the techniques were defined as described in the Methods section “Cell-free DNA SNV detection error rate.” In total, we generated 10,000 simulated data sets for each patient in each scenario, which translates to 2,040,000 (10,000 × 51 (TF) × 4 (technique)) simulated data sets per patient or 3,060,000,000 (2,040,000 × 150) simulated data sets in total. Using the simulated sample with a TF of 0, we could obtain a “background noise” profile for each patient. For each patient, a confident lowest detectable TF is defined by >95% true positive rate (TPR, i.e., we performed in silico sequencing of this patient 10,000 times, and we detected the tumor presence more than 95% of the time) and >68% true negative rate (TNR, i.e., we performed in silico sequencing of samples with TF = 0 10,000 times, and more than 68% of the time we do not detect cancer presence).

### Generative AI and AI-assisted technologies in the writing process

During the preparation of this work, the authors used chatGPT (https://chatgpt.com/) in order to rephrase sentences. After using this tool, the authors reviewed and edited the content as needed and take full responsibility for the content of the publication.

### Software availability

The algorithm and code for conducting analyses in this manuscript is available at GitHub (https://github.com/UMCUGenetics/NanoRCS/) and as Supplemental Code.

## Data access

All raw and processed sequencing data generated in this study have been submitted to the European Genome-Phenome Archive (EGA; https://web2.ega-archive.org/) under accession number EGAS50000000154 and EGAS50000000695. Raw AFM images were uploaded to Zenodo at doi.org/10.5281/zenodo.10423114 (HC03), doi.org/10.5281/zenodo.10423541 (OVCA01), doi.org/10.5281/zenodo.10423356(OVCA07), and doi.org/10.5281/zenodo.10423726 (DNA ladder) with open access.

## Supplemental Material

Supplement 1

Supplement 2

Supplement 3

Supplement 4

Supplement 5

Supplement 6

Supplement 7

Supplement 8

Supplement 9

Supplement 10

Supplement 11

Supplement 12

Supplement 13

Supplement 14

Supplement 15

Supplement 16

Supplement 17

Supplement 18

Supplement 19

## References

[GR279144CHEC1] Acevedo A, Brodsky L, Andino R. 2014. Mutational and fitness landscapes of an RNA virus revealed through population sequencing. Nature 505: 686–690. 10.1038/nature12861 24284629 PMC4111796

[GR279144CHEC2] Adalsteinsson VA, Ha G, Freeman SS, Choudhury AD, Stover DG, Parsons HA, Gydush G, Reed SC, Rotem D, Rhoades J, 2017. Scalable whole-exome sequencing of cell-free DNA reveals high concordance with metastatic tumors. Nat Commun 8: 1324. 10.1038/s41467-017-00965-y 29109393 PMC5673918

[GR279144CHEC3] Alborelli I, Generali D, Jermann P, Cappelletti MR, Ferrero G, Scaggiante B, Bortul M, Zanconati F, Nicolet S, Haegele J, 2019. Cell-free DNA analysis in healthy individuals by next-generation sequencing: a proof of concept and technical validation study. Cell Death Dis 10: 534. 10.1038/s41419-019-1770-3 31296838 PMC6624284

[GR279144CHEC4] Arslan S, Garcia FJ, Guo M, Kellinger MW, Kruglyak S, LeVieux JA, Mah AH, Wang H, Zhao J, Zhou C, 2024. Sequencing by avidity enables high accuracy with low reagent consumption. Nat Biotechnol 42: 132–138. 10.1038/s41587-023-01750-7 37231263 PMC10791576

[GR279144CHEC5] Bettegowda C, Sausen M, Leary RJ, Kinde I, Wang Y, Agrawal N, Bartlett BR, Wang H, Luber B, Alani RM, 2014. Detection of circulating tumor DNA in early- and late-stage human malignancies. Sci Transl Med 6: a24. 10.1126/scitranslmed.3007094 PMC4017867 24553385

[GR279144CHEC6] Bielski CM, Zehir A, Penson AV, Donoghue MTA, Chatila W, Armenia J, Chang MT, Schram AM, Jonsson P, Bandlamudi C, 2018. Genome doubling shapes the evolution and prognosis of advanced cancers. Nat Genet 50: 1189–1195. 10.1038/s41588-018-0165-1 30013179 PMC6072608

[GR279144CHEC7] Binnig G, Quate CF, Gerber C. 1986. Atomic force microscope. Phys Rev Lett 56: 930–933. 10.1103/PhysRevLett.56.930 10033323

[GR279144CHEC8] Bronkhorst AJ, Ungerer V, Holdenrieder S. 2019. The emerging role of cell-free DNA as a molecular marker for cancer management. Biomol Detect Quantif 17: 100087. 10.1016/j.bdq.2019.100087 30923679 PMC6425120

[GR279144CHEC9] Burnham P, Dadhania D, Heyang M, Chen F, Westblade LF, Suthanthiran M, Lee JR, De Vlaminck I. 2018. Urinary cell-free DNA is a versatile analyte for monitoring infections of the urinary tract. Nat Commun 9: 2412. 10.1038/s41467-018-04745-0 29925834 PMC6010457

[GR279144CHEC10] Bushnell B. 2024. BBMap. https://www.sourceforge.net/projects/bbmap/ (Accessed Oct 31, 2024).

[GR279144CHEC68] The Cancer Genome Atlas Research Network. 2011. Integrated genomic analyses of ovarian carcinoma. Nature 474: 609–615. 10.1038/nature10166 21720365 PMC3163504

[GR279144CHEC69] The Cancer Genome Atlas Research Network. 2017. Integrated genomic characterization of oesophageal carcinoma. Nature 541: 169–175. 10.1038/nature20805 28052061 PMC5651175

[GR279144CHEC11] The Cancer Genome Atlas Research Network, Weinstein JN, Collisson EA, Mills GB, Shaw KRM, Ozenberger BA, Ellrott K, Shmulevich I, Sander C, Stuart JM. 2013. The Cancer Genome Atlas Pan-Cancer analysis project. Nat Genet 45: 1113–1120. 10.1038/ng.2764 24071849 PMC3919969

[GR279144CHEC12] Chen S. 2023. Ultrafast one-pass FASTQ data preprocessing, quality control, and deduplication using fastp. Imeta 2: e107. 10.1002/imt2.107 38868435 PMC10989850

[GR279144CHEC13] Chen E, Cario CL, Leong L, Lopez K, Márquez CP, Chu C, Li PS, Oropeza E, Tenggara I, Cowan J, 2021. Cell-free DNA concentration and fragment size as a biomarker for prostate cancer. Sci Rep 11: 5040. 10.1038/s41598-021-84507-z 33658587 PMC7930042

[GR279144CHEC14] Cheng Z, Mirza H, Ennis DP, Smith P, Morrill Gavarró L, Sokota C, Giannone G, Goranova T, Bradley T, Piskorz A, 2022. The genomic landscape of early-stage ovarian high-grade serous carcinoma. Clin Cancer Res 28: 2911–2922. 10.1158/1078-0432.CCR-21-1643 35398881 PMC7612959

[GR279144CHEC15] Choy LYL, Peng W, Jiang P, Cheng SH, Yu SCY, Shang H, Olivia Tse OY, Wong J, Wong VWS, Wong GLH, 2022. Single-molecule sequencing enables long cell-free DNA detection and direct methylation analysis for cancer patients. Clin Chem 68: 1151–1163. 10.1093/clinchem/hvac086 35587130

[GR279144CHEC16] Corcoran RB, Chabner BA. 2018. Application of cell-free DNA analysis to cancer treatment. N Engl J Med 379: 1754–1765. 10.1056/NEJMra1706174 30380390

[GR279144CHEC17] Cristiano S, Leal A, Phallen J, Fiksel J, Adleff V, Bruhm DC, Jensen SØ, Medina JE, Hruban C, White JR, 2019. Genome-wide cell-free DNA fragmentation in patients with cancer. Nature 570: 385–389. 10.1038/s41586-019-1272-6 31142840 PMC6774252

[GR279144CHEC18] Delahaye C, Nicolas J. 2021. Sequencing DNA with nanopores: troubles and biases. PLoS One 16: e0257521. 10.1371/journal.pone.0257521 34597327 PMC8486125

[GR279144CHEC19] Deng DZQ, Verhage J, Neudorf C, Corbett-Detig R, Mekonen H, Castaldi PJ, Vollmers C. 2024. R2c2 + UMI: combining concatemeric and unique molecular identifier-based consensus sequencing enables ultra-accurate sequencing of amplicons on Oxford Nanopore Technologies sequencers. PNAS Nexus 3: gae336. 10.1093/pnasnexus/pgae336 PMC11376274 39238604

[GR279144CHEC20] de Witte CJ, Espejo Valle-Inclan J, Hami N, Lõhmussaar K, Kopper O, Vreuls CPH, Jonges GN, van Diest P, Nguyen L, Clevers H, 2020. Patient-derived ovarian cancer organoids mimic clinical response and exhibit heterogeneous inter- and intrapatient drug responses. Cell Rep 31: 107762. 10.1016/j.celrep.2020.107762 32553164

[GR279144CHEC21] de Witte CJ, Kutzera J, van Hoeck A, Nguyen L, Boere IA, Jalving M, Ottevanger PB, van Schaik-van de Mheen C, Stevense M, Kloosterman WP, 2022. Distinct genomic profiles are associated with treatment response and survival in ovarian cancer. Cancers (Basel) 14: 1511. 10.3390/cancers14061511 35326660 PMC8946149

[GR279144CHEC22] Diaz LA Jr, Bardelli A. 2014. Liquid biopsies: genotyping circulating tumor DNA. J Clin Oncol 32: 579–586. 10.1200/JCO.2012.45.2011 24449238 PMC4820760

[GR279144CHEC23] Do H, Dobrovic A. 2015. Sequence artifacts in DNA from formalin-fixed tissues: causes and strategies for minimization. Clin Chem 61: 64–71. 10.1373/clinchem.2014.223040 25421801

[GR279144CHEC24] Ewels PA, Peltzer A, Fillinger S, Patel H, Alneberg J, Wilm A, Garcia MU, Di Tommaso P, Nahnsen S. 2020. The nf-core framework for community-curated bioinformatics pipelines. Nat Biotechnol 38: 276–278. 10.1038/s41587-020-0439-x 32055031

[GR279144CHEC25] Frankell AM, Jammula S, Li X, Contino G, Killcoyne S, Abbas S, Perner J, Bower L, Devonshire G, Ococks E, 2019. The landscape of selection in 551 esophageal adenocarcinomas defines genomic biomarkers for the clinic. Nat Genet 51: 506–516. 10.1038/s41588-018-0331-5 30718927 PMC6420087

[GR279144CHEC26] Groeneweg JW, Roze JF, Peters EDJ, Sereno F, Brink AGJ, Paijens ST, Nijman HW, van Meurs HS, van Lonkhuijzen LRCW, Piek JMJ, 2021. *FOXL2* and *TERT* promoter mutation detection in circulating tumor DNA of adult granulosa cell tumors as biomarker for disease monitoring. Gynecol Oncol 162: 413–420. 10.1016/j.ygyno.2021.05.027 34083028

[GR279144CHEC27] Hanssen F, Garcia MU, Folkersen L, Pedersen AS, Lescai F, Jodoin S, Miller E, Seybold M, Wacker O, Smith N, 2024. Scalable and efficient DNA sequencing analysis on different compute infrastructures aiding variant discovery. NAR Genom Bioinform 6: lqae031. 10.1093/nargab/lqae031 38666213 PMC11044436

[GR279144CHEC28] Harris AR, Raveendran M, Worley KC, Rogers J. 2020. Unusual sequence characteristics of human chromosome 19 are conserved across 11 nonhuman primates. BMC Evol Biol 20: 33. 10.1186/s12862-020-1595-9 32106815 PMC7045612

[GR279144CHEC29] Hieronymus H, Murali R, Tin A, Yadav K, Abida W, Moller H, Berney D, Scher H, Carver B, Scardino P, 2018. Tumor copy number alteration burden is a pan-cancer prognostic factor associated with recurrence and death. eLife 7: e37294. 10.7554/eLife.37294 30178746 PMC6145837

[GR279144CHEC30] Hoadley KA, Yau C, Hinoue T, Wolf DM, Lazar AJ, Drill E, Shen R, Taylor AM, Cherniack AD, Thorsson V, 2018. Cell-of-origin patterns dominate the molecular classification of 10,000 tumors from 33 types of cancer. Cell 173: 291–304.e6. 10.1016/j.cell.2018.03.022 29625048 PMC5957518

[GR279144CHEC31] Hon T, Mars K, Young G, Tsai Y-C, Karalius JW, Landolin JM, Maurer N, Kudrna D, Hardigan MA, Steiner CC, 2020. Highly accurate long-read HiFi sequencing data for five complex genomes. Sci Data 7: 399. 10.1038/s41597-020-00743-4 33203859 PMC7673114

[GR279144CHEC32] Hu Y, Ulrich BC, Supplee J, Kuang Y, Lizotte PH, Feeney NB, Guibert NM, Awad MM, Wong K-K, Jänne PA, 2018. False-positive plasma genotyping due to clonal hematopoiesis. Clin Cancer Res 24: 4437–4443. 10.1158/1078-0432.CCR-18-0143 29567812

[GR279144CHEC33] Husain H, Nykin D, Bui N, Quan D, Gomez G, Woodward B, Venkatapathy S, Duttagupta R, Fung E, Lippman SM, 2017. Cell-free DNA from ascites and pleural effusions: molecular insights into genomic aberrations and disease biology. Mol Cancer Ther 16: 948–955. 10.1158/1535-7163.MCT-16-0436 28468865

[GR279144CHEC34] Husain H, Pavlick DC, Fendler BJ, Madison RW, Decker B, Gjoerup O, Parachoniak CA, McLaughlin-Drubin M, Erlich RL, Schrock AB, 2022. Tumor fraction correlates with detection of actionable variants across > 23,000 circulating tumor DNA samples. JCO Precis Oncol 6: e2200261. 10.1200/PO.22.00261 36265119 PMC9616642

[GR279144CHEC70] The ICGC/TCGA Pan-Cancer Analysis of Whole Genomes Consortium. 2020. Pan-cancer analysis of whole genomes. Nature 578: 82–93. 10.1038/s41586-020-1969-6 32025007 PMC7025898

[GR279144CHEC35] Karst SM, Ziels RM, Kirkegaard RH, Sørensen EA, McDonald D, Zhu Q, Knight R, Albertsen M. 2021. High-accuracy long-read amplicon sequences using unique molecular identifiers with Nanopore or PacBio sequencing. Nat Methods 18: 165–169. 10.1038/s41592-020-01041-y 33432244

[GR279144CHEC36] Kivioja T, Vähärautio A, Karlsson K, Bonke M, Enge M, Linnarsson S, Taipale J. 2012. Counting absolute numbers of molecules using unique molecular identifiers. Nat Methods 9: 72–74. 10.1038/nmeth.1778 22101854

[GR279144CHEC37] Kopper O, de Witte CJ, Lõhmussaar K, Valle-Inclan JE, Hami N, Kester L, Balgobind AV, Korving J, Proost N, Begthel H, 2019. An organoid platform for ovarian cancer captures intra- and interpatient heterogeneity. Nat Med 25: 838–849. 10.1038/s41591-019-0422-6 31011202

[GR279144CHEC38] Lau BT, Almeda A, Schauer M, McNamara M, Bai X, Meng Q, Partha M, Grimes SM, Lee H, Heestand GM, 2023. Single-molecule methylation profiles of cell-free DNA in cancer with nanopore sequencing. Genome Med 15: 33. 10.1186/s13073-023-01178-3 37138315 PMC10155347

[GR279144CHEC39] Lee DD, Seung HS. 1999. Learning the parts of objects by non-negative matrix factorization. Nature 401: 788–791. 10.1038/44565 10548103

[GR279144CHEC40] Li H. 2018. Minimap2: pairwise alignment for nucleotide sequences. Bioinformatics 34: 3094–3100. 10.1093/bioinformatics/bty191 29750242 PMC6137996

[GR279144CHEC41] Li C, Chng KR, Boey EJH, Ng AHQ, Wilm A, Nagarajan N. 2016. INC-seq: accurate single molecule reads using nanopore sequencing. GigaScience 5: 34. 10.1186/s13742-016-0140-7 27485345 PMC4970289

[GR279144CHEC42] Liu Y. 2022. At the dawn: cell-free DNA fragmentomics and gene regulation. Br J Cancer 126: 379–390. 10.1038/s41416-021-01635-z 34815523 PMC8810841

[GR279144CHEC43] Lo YMD, Han DSC, Jiang P, Chiu RWK. 2021. Epigenetics, fragmentomics, and topology of cell-free DNA in liquid biopsies. Science 372: eaaw3616. 10.1126/science.aaw3616 33833097

[GR279144CHEC44] Lou DI, Hussmann JA, McBee RM, Acevedo A, Andino R, Press WH, Sawyer SL. 2013. High-throughput DNA sequencing errors are reduced by orders of magnitude using circle sequencing. Proc Natl Acad Sci 110: 19872–19877. 10.1073/pnas.1319590110 24243955 PMC3856802

[GR279144CHEC45] Lustberg MB, Stover DG, Chalmers JJ. 2018. Implementing liquid biopsies in clinical trials: state of affairs, opportunities, and challenges. Cancer J 24: 61–64. 10.1097/PPO.0000000000000309 29601331 PMC5880324

[GR279144CHEC46] Marcozzi A, Jager M, Elferink M, Straver R, van Ginkel JH, Peltenburg B, Chen L-T, Renkens I, van Kuik J, Terhaard C, 2021. Accurate detection of circulating tumor DNA using nanopore consensus sequencing. NPJ Genom Med 6: 106. 10.1038/s41525-021-00272-y 34887408 PMC8660781

[GR279144CHEC47] Martínez-Jiménez F, Movasati A, Brunner SR, Nguyen L, Priestley P, Cuppen E, Van Hoeck A. 2023. Pan-cancer whole-genome comparison of primary and metastatic solid tumours. Nature 618: 333–341. 10.1038/s41586-023-06054-z 37165194 PMC10247378

[GR279144CHEC48] McEwen AE, Leary SES, Lockwood CM. 2020. Beyond the blood: CSF-derived cfDNA for diagnosis and characterization of CNS tumors. Front Cell Dev Biol 8: 45. 10.3389/fcell.2020.00045 32133357 PMC7039816

[GR279144CHEC49] Moldovan N, van der Pol Y, van den Ende T, Boers D, Verkuijlen S, Creemers A, Ramaker J, Vu T, Bootsma S, Lenos KJ, 2024. Multi-modal cell-free DNA genomic and fragmentomic patterns enhance cancer survival and recurrence analysis. Cell Rep Med 5: 101349. 10.1016/j.xcrm.2023.101349 38128532 PMC10829758

[GR279144CHEC50] Mouliere F, El Messaoudi S, Pang D, Dritschilo A, Thierry AR. 2014. Multi-marker analysis of circulating cell-free DNA toward personalized medicine for colorectal cancer. Mol Oncol 8: 927–941. 10.1016/j.molonc.2014.02.005 24698732 PMC5528519

[GR279144CHEC51] Mouliere F, Chandrananda D, Piskorz AM, Moore EK, Morris J, Ahlborn LB, Mair R, Goranova T, Marass F, Heider K, 2018. Enhanced detection of circulating tumor DNA by fragment size analysis. Sci Transl Med 10: eaat4921. 10.1126/scitranslmed.aat4921 30404863 PMC6483061

[GR279144CHEC52] Nawroz H, Koch W, Anker P, Stroun M, Sidransky D. 1996. Microsatellite alterations in serum DNA of head and neck cancer patients. Nat Med 2: 1035–1037. 10.1038/nm0996-1035 8782464

[GR279144CHEC53] Nones K, Waddell N, Wayte N, Patch AM, Bailey P, Newell F, Holmes O, Fink JL, Quinn MCJ, Tang YH, 2014. Genomic catastrophes frequently arise in esophageal adenocarcinoma and drive tumorigenesis. Nat Commun 5: 5224. 10.1038/ncomms6224 25351503 PMC4596003

[GR279144CHEC54] Peneder P, Stütz AM, Surdez D, Krumbholz M, Semper S, Chicard M, Sheffield NC, Pierron G, Lapouble E, Tötzl M, 2021. Multimodal analysis of cell-free DNA whole-genome sequencing for pediatric cancers with low mutational burden. Nat Commun 12: 3230. 10.1038/s41467-021-23445-w 34050156 PMC8163828

[GR279144CHEC55] Phallen J, Sausen M, Adleff V, Leal A, Hruban C, White J, Anagnostou V, Fiksel J, Cristiano S, Papp E, 2017. Direct detection of early-stage cancers using circulating tumor DNA. Sci Transl Med 9: eaan2415. 10.1126/scitranslmed.aan2415 28814544 PMC6714979

[GR279144CHEC56] Quinlan AR, Hall IM. 2010. BEDTools: a flexible suite of utilities for comparing genomic features. Bioinformatics 26: 841–842. 10.1093/bioinformatics/btq033 20110278 PMC2832824

[GR279144CHEC57] Razavi P, Li BT, Brown DN, Jung B, Hubbell E, Shen R, Abida W, Juluru K, De Bruijn I, Hou C, 2019. High-intensity sequencing reveals the sources of plasma circulating cell-free DNA variants. Nat Med 25: 1928–1937. 10.1038/s41591-019-0652-7 31768066 PMC7061455

[GR279144CHEC58] Renaud G, Nørgaard M, Lindberg J, Grönberg H, De Laere B, Jensen JB, Borre M, Andersen CL, Sørensen KD, Maretty L, 2022. Unsupervised detection of fragment length signatures of circulating tumor DNA using non-negative matrix factorization. eLife 11: e71569. 10.7554/eLife.71569 35894300 PMC9363120

[GR279144CHEC59] Robbe P, Popitsch N, Knight SJL, Antoniou P, Becq J, He M, Kanapin A, Samsonova A, Vavoulis DV, Ross MT, 2018. Clinical whole-genome sequencing from routine formalin-fixed, paraffin-embedded specimens: pilot study for the 100,000 Genomes Project. Genet Med 20: 1196–1205. 10.1038/gim.2017.241 29388947 PMC6520241

[GR279144CHEC60] Roerink SF, Sasaki N, Lee-Six H, Young MD, Alexandrov LB, Behjati S, Mitchell TJ, Grossmann S, Lightfoot H, Egan DA, 2018. Intra-tumour diversification in colorectal cancer at the single-cell level. Nature 556: 457–462. 10.1038/s41586-018-0024-3 29643510

[GR279144CHEC61] Roze J, Monroe G, Kutzera J, Groeneweg J, Stelloo E, Paijens S, Nijman H, van Meurs H, van Lonkhuijzen L, Piek J, 2020. Whole genome analysis of ovarian granulosa cell tumors reveals tumor heterogeneity and a high-grade TP53-specific subgroup. Cancers (Basel) 12: 1308. 10.3390/cancers12051308 32455687 PMC7281495

[GR279144CHEC62] Saunders CT, Wong WSW, Swamy S, Becq J, Murray LJ, Cheetham RK. 2012. Strelka: accurate somatic small-variant calling from sequenced tumor-normal sample pairs. Bioinformatics 28: 1811–1817. 10.1093/bioinformatics/bts271 22581179

[GR279144CHEC63] Simpson JT, Workman RE, Zuzarte PC, David M, Dursi LJ, Timp W. 2017. Detecting DNA cytosine methylation using nanopore sequencing. Nat Methods 14: 407–410. 10.1038/nmeth.4184 28218898

[GR279144CHEC64] Smith P, Bradley T, Gavarró LM, Goranova T, Ennis DP, Mirza HB, De Silva D, Piskorz AM, Sauer CM, Al-Khalidi S, 2023. The copy number and mutational landscape of recurrent ovarian high-grade serous carcinoma. Nat Commun 14: 4387. 10.1038/s41467-023-39867-7 37474499 PMC10359414

[GR279144CHEC65] Steele CD, Abbasi A, Islam SMA, Bowes AL, Khandekar A, Haase K, Hames-Fathi S, Ajayi D, Verfaillie A, Dhami P, 2022. Signatures of copy number alterations in human cancer. Nature 606: 984–991. 10.1038/s41586-022-04738-6 35705804 PMC9242861

[GR279144CHEC66] Stoler N, Nekrutenko A. 2021. Sequencing error profiles of Illumina sequencing instruments. NAR Genom Bioinform 3: lqab019. 10.1093/nargab/lqab019 33817639 PMC8002175

[GR279144CHEC67] Stroun M, Anker P, Lyautey J, Lederrey C, Maurice PA. 1987. Isolation and characterization of DNA from the plasma of cancer patients. Eur J Cancer Clin Oncol 23: 707–712. 10.1016/0277-5379(87)90266-5 3653190

[GR279144CHEC71] Thirunavukarasu D, Cheng LY, Song P, Chen SX, Borad MJ, Kwong L, James P, Turner DJ, Zhang DY. 2021. Oncogene Concatenated Enriched Amplicon Nanopore Sequencing for rapid, accurate, and affordable somatic mutation detection. Genome Biol 22: 227. 10.1186/s13059-021-02449-1 34482832 PMC8419911

[GR279144CHEC72] Udomruk S, Orrapin S, Pruksakorn D, Chaiyawat P. 2021. Size distribution of cell-free DNA in oncology. Crit Rev Oncol Hematol 166: 103455. 10.1016/j.critrevonc.2021.103455 34464717

[GR279144CHEC73] Van der Auwera GA, O'Connor BD. 2020. Genomics in the cloud: using Docker, GATK, and WDL in Terra. O'Reilly Media, Sebastopol, CA.

[GR279144CHEC74] van der Pol Y, Mouliere F. 2019. Toward the early detection of cancer by decoding the epigenetic and environmental fingerprints of cell-free DNA. Cancer Cell 36: 350–368. 10.1016/j.ccell.2019.09.003 31614115

[GR279144CHEC75] van der Pol Y, Tantyo NA, Evander N, Hentschel AE, Wever BMM, Ramaker J, Bootsma S, Fransen MF, Lenos KJ, Vermeulen L, 2023. Real-time analysis of the cancer genome and fragmentome from plasma and urine cell-free DNA using nanopore sequencing. EMBO Mol Med 15: e17282. 10.15252/emmm.202217282 37942753 PMC10701599

[GR279144CHEC76] Vermeulen C, Pagès-Gallego M, Kester L, Kranendonk MEG, Wesseling P, Verburg N, de Witt Hamer P, Kooi EJ, Dankmeijer L, van der Lugt J, 2023. Ultra-fast deep-learned CNS tumour classification during surgery. Nature 622: 842–849. 10.1038/s41586-023-06615-2 37821699 PMC10600004

[GR279144CHEC77] Volden R, Palmer T, Byrne A, Cole C, Schmitz RJ, Green RE, Vollmers C. 2018. Improving nanopore read accuracy with the R2C2 method enables the sequencing of highly multiplexed full-length single-cell cDNA. Proc Natl Acad Sci 115: 9726–9731. 10.1073/pnas.1806447115 30201725 PMC6166824

[GR279144CHEC78] Wan JCM, Massie C, Garcia-Corbacho J, Mouliere F, Brenton JD, Caldas C, Pacey S, Baird R, Rosenfeld N. 2017. Liquid biopsies come of age: towards implementation of circulating tumour DNA. Nat Rev Cancer 17: 223–238. 10.1038/nrc.2017.7 28233803

[GR279144CHEC79] Weilguny L, De Maio N, Munro R, Manser C, Birney E, Loose M, Goldman N. 2023. Dynamic, adaptive sampling during nanopore sequencing using Bayesian experimental design. Nat Biotechnol 41: 1018–1025. 10.1038/s41587-022-01580-z 36593407 PMC10344778

[GR279144CHEC80] Werner B, Yuwono N, Duggan J, Liu D, David C, Srirangan S, Provan P, INOVATe Investigators, DeFazio A, Arora V, 2021. Cell-free DNA is abundant in ascites and represents a liquid biopsy of ovarian cancer. Gynecol Oncol 162: 720–727. 10.1016/j.ygyno.2021.06.028 34454680

[GR279144CHEC81] Wilson BD, Eisenstein M, Soh HT. 2019. High-fidelity nanopore sequencing of ultra-short DNA targets. Anal Chem 91: 6783–6789. 10.1021/acs.analchem.9b00856 31038923 PMC6533607

[GR279144CHEC82] Yang SYC, Pugh TJ, Oza AM. 2022. Double trouble: whole-genome doubling distinguishes early from late ovarian cancer. Clin Cancer Res 28: 2730–2732. 10.1158/1078-0432.CCR-22-0336 35476137 PMC9306310

[GR279144CHEC83] Yu SCY, Jiang P, Peng W, Cheng SH, Cheung YTT, Tse OYO, Shang H, Poon LC, Leung TY, Chan KCA, 2021. Single-molecule sequencing reveals a large population of long cell-free DNA molecules in maternal plasma. Proc Natl Acad Sci 118: e2114937118. 10.1073/pnas.2114937118 34873045 PMC8685924

[GR279144CHEC84] Zviran A, Schulman RC, Shah M, Hill STK, Deochand S, Khamnei CC, Maloney D, Patel K, Liao W, Widman AJ, 2020. Genome-wide cell-free DNA mutational integration enables ultra-sensitive cancer monitoring. Nat Med 26: 1114–1124. 10.1038/s41591-020-0915-3 32483360 PMC8108131

