# Supplementary material for: Nanopore-based consensus sequencing enables accurate multimodal tumor cell-free DNA profiling
Source: Genome Res. 2025 Apr;35(4):886–99. doi: 10.1101/gr.279144.124 (PMC12047234; doi:10.1101/gr.279144.124)
Supplement: Supplement 1 [file Supplemental_Fig_S1.pdf]

Supplemental Figures

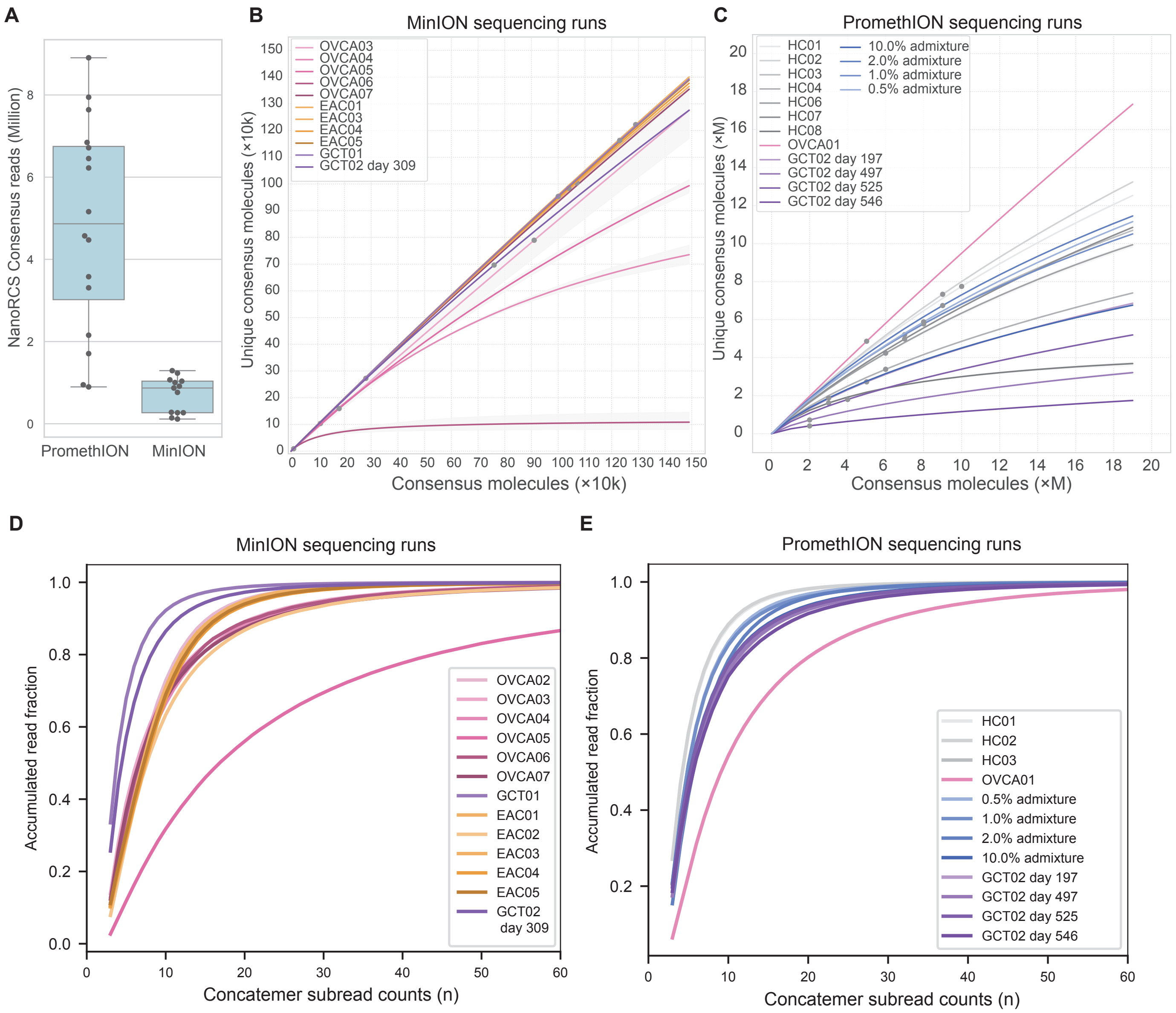

Supplemental Figure S1. Throughput of NanoRCS and consensus-forming subread counts using PromethION and MinION. (A) Consensus read counts for PromethION and MinION NanoRCS libraries. (B-C) Library complexity estimation for all (B) MinION and (C) PromethION runs. gray dots indicate the sequenced consensus molecules in each sample, solid lines indicate the estimated number of unique DNA molecules that could be acquired according to the complexity of sequenced consensus sequences. Lightgray shaded area indicates the 95% confidence interval of the variability. In PromethION runs, the confidence intervals were too narrow to be observed in the plot. Library complexity estimation failed for OVCA02. xM, million reads. x10k, ten thousand reads.(D) Distribution of subread counts forming consensus reads in samples sequenced with MinION. Accumulated read fraction is shown on the y-axis to indicate that the median subread counts is between n=3-17. (E) Distribution of subread counts forming consensus reads in samples sequenced with PromethION. Accumulated read fraction is shown on the y-axis to indicate that the median subread counts is between n=4-9.
