# Supplementary material for: Nanopore-based consensus sequencing enables accurate multimodal tumor cell-free DNA profiling
Source: Genome Res. 2025 Apr;35(4):886–99. doi: 10.1101/gr.279144.124 (PMC12047234; doi:10.1101/gr.279144.124)
Supplement: Supplement 2 [file Supplemental_Fig_S2.pdf]

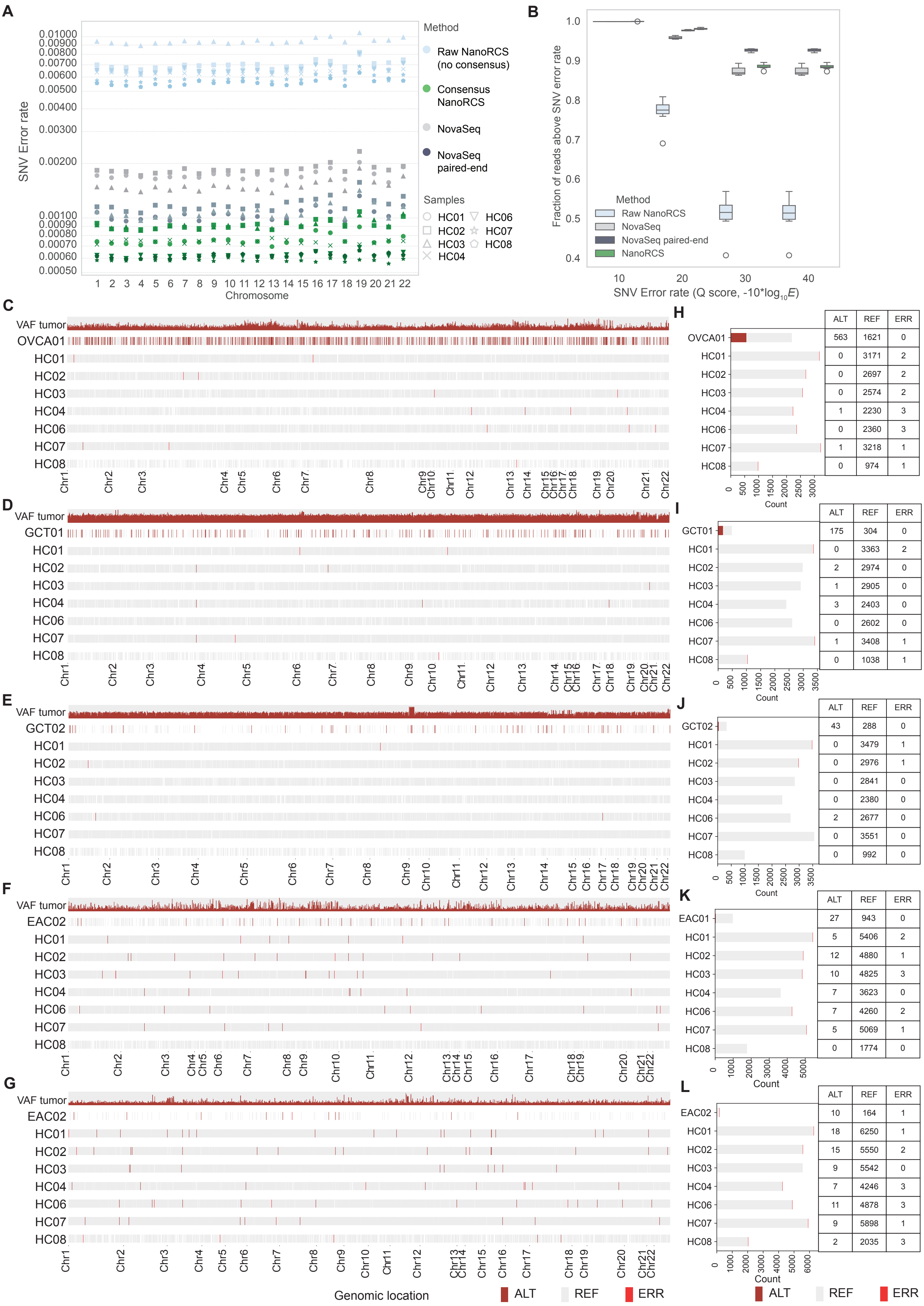

H

	ALT	REF	ERR
OVCA01	563	1621	0
HC01	0	3171	2
HC02	0	2697	2
HC03	0	2574	2
HC04	1	2230	3
HC06	0	2360	3
HC07	1	3218	1
HC08	0	974	1

I

	ALT	REF	ERR
GCT01	175	304	0
HC01	0	3363	2
HC02	2	2974	0
HC03	1	2905	0
HC04	3	2403	0
HC06	0	2602	0
HC07	1	3408	1
HC08	0	1038	1

J

	ALT	REF	ERR
GCT02	43	288	0
HC01	0	3479	1
HC02	0	2976	1
HC03	0	2841	0
HC04	0	2380	0
HC06	2	2677	0
HC07	0	3551	0
HC08	0	992	0

K

	ALT	REF	ERR
EAC01	27	943	0
HC01	5	5406	2
HC02	12	4880	1
HC03	10	4825	3
HC04	7	3623	0
HC06	7	4260	2
HC07	5	5069	1
HC08	0	1774	0

L

	ALT	REF	ERR
EAC02	10	164	1
HC01	18	6250	1
HC02	15	5550	2
HC03	9	5542	0
HC04	7	4246	3
HC06	11	4878	3
HC07	9	5898	1
HC08	2	2035	3

Supplementary Figure S2. NanoRCS enables confident SNV detection on Nanopore.
(A) Single-nucleotide error rate per chromosome in cfDNA of seven healthy controls using different sequencing methods. (B) Fraction of reads with read quality of at least phred score of 30, 40, 50, 60 (equivalent to SNV error rate of 1 in 103, 1 in 104, 1 in 105 bases) in different sequencing methods. (C-G) SNV observations in the five liquid biopsy samples with known tumor somatic SNV profile. For each panel, the top row shows the VAF of detected mutations in the tumor biopsy, the second row represents the MUT or REF allele observations in the liquid biopsy of the corresponding patient, and the bottom seven rows represent the observations in seven healthy controls (not downsampled). (H-L) Raw counts of SNV observations corresponding to the data shown in (C-G).
