# Supplementary material for: Nanopore-based consensus sequencing enables accurate multimodal tumor cell-free DNA profiling
Source: Genome Res. 2025 Apr;35(4):886–99. doi: 10.1101/gr.279144.124 (PMC12047234; doi:10.1101/gr.279144.124)
Supplement: Supplement 3 [file Supplemental_Fig_S3.pdf]

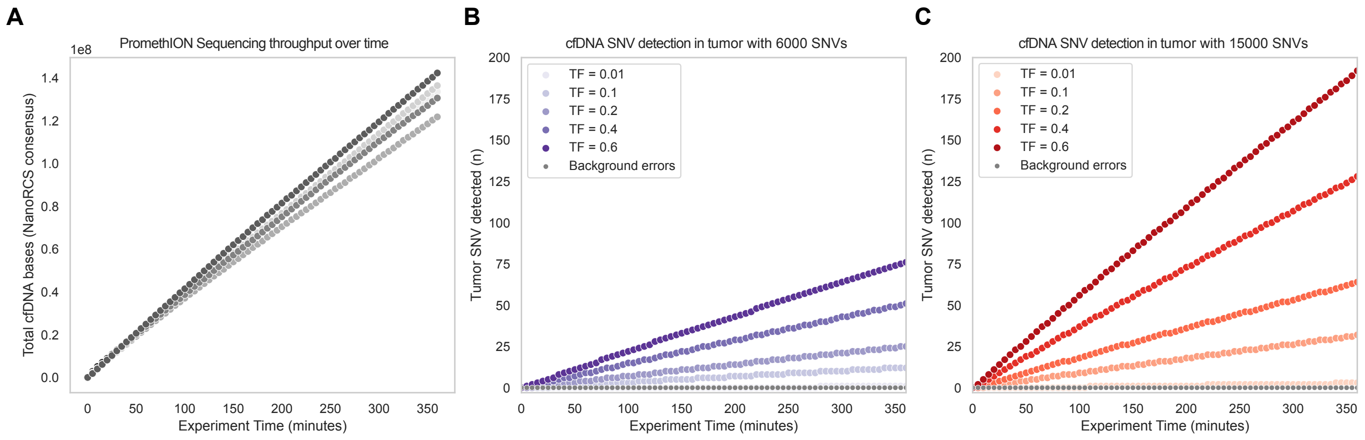

Supplemental Figure S3. Simulation of tumor-specific SNVs detection with NanoRCS over time across different tumor fractions. (A) The accumulation of cfDNA (NanoRCS consensus) bases sequenced over time (minutes) of six selected PromethION sequencing runs. (B-C) Tumor-specific SNVs over time across five different TFs: 0.01, 0.1, 0.2, 0.4, and 0.6, in comparison with background errors in tumor with (B) 6000 and (C) 15000 tumor-specific SNVs. Tumor fractions greater than 0.1 are detectable within 20 to 110 minutes of experiment time, with higher tumor fractions being detected sooner. For TF at 0.01, with 6000 and 15000 tumor-specific SNVs, 280 and 110 minutes is required to distinguish from background errors, respectively.
