# Supplementary material for: Nanopore-based consensus sequencing enables accurate multimodal tumor cell-free DNA profiling
Source: Genome Res. 2025 Apr;35(4):886–99. doi: 10.1101/gr.279144.124 (PMC12047234; doi:10.1101/gr.279144.124)
Supplement: Supplement 4 [file Supplemental_Fig_S4.pdf]

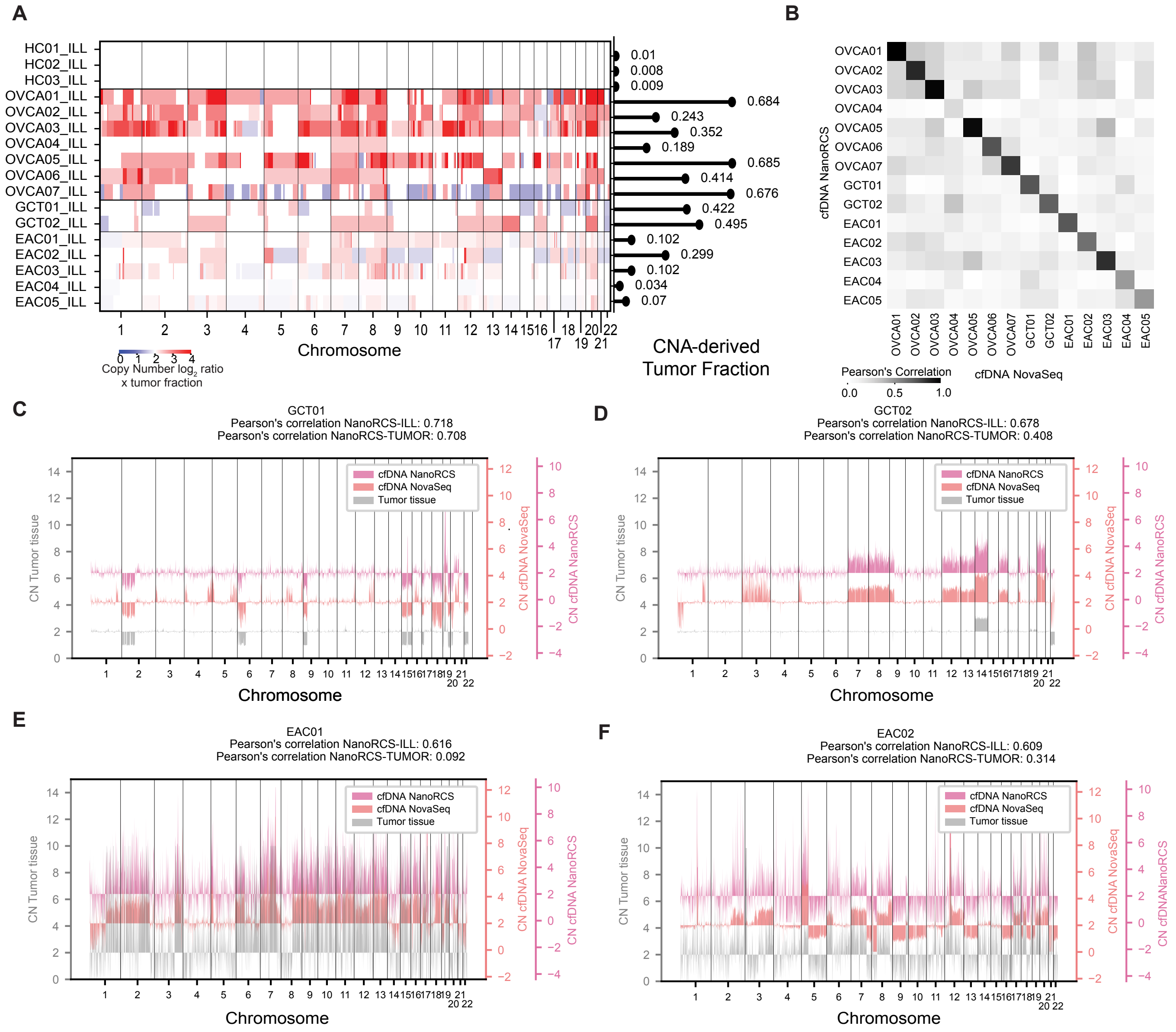

Supplemental Figure S4. Comparison of copy number alterations in NanoRCS and NovaSeq cfDNA and tumor biopsy whole-genome sequencing. (A) Copy number alterations (CNAs) and derived tumor fraction (lollipops) for cfDNA samples obtained using NovaSeq across different sample types (HC, healthy controls; OVCA, ovarian carcinoma; GCT, granulosa cell tumor; EAC, esophageal adenocarcinoma). Red indicates copy number gain and blue indicates copy number loss. Color intensity indicates the copy number alteration multiplied by the tumor fraction in cfDNA. (B) Correlation of CNA profiles in NanoRCS cfDNA sequencing and NovaSeq cfDNA sequencing. Color intensity indicates Pearson's correlation. (C-F) Comparison of genome-wide copy numbers for NanoRCS (pink) and NovaSeq (orange) cfDNA sequencing and whole-genome tumor biopsy sequencing (gray) in samples (C) GCT01, (D) GCT02, (E) EAC01 and (F) EAC02 of which we have matching tumor sequencing data. Pearson's correlations between NanoRCS (NRCS) and NovaSeq (ILL) and between NanoRCS (NRCS) and tumor biopsy (TUMOR) are indicated above each graph. All CNA data was generated using ichorCNA.
