# Supplementary material for: Nanopore-based consensus sequencing enables accurate multimodal tumor cell-free DNA profiling
Source: Genome Res. 2025 Apr;35(4):886–99. doi: 10.1101/gr.279144.124 (PMC12047234; doi:10.1101/gr.279144.124)
Supplement: Supplement 5 [file Supplemental_Fig_S5.pdf]

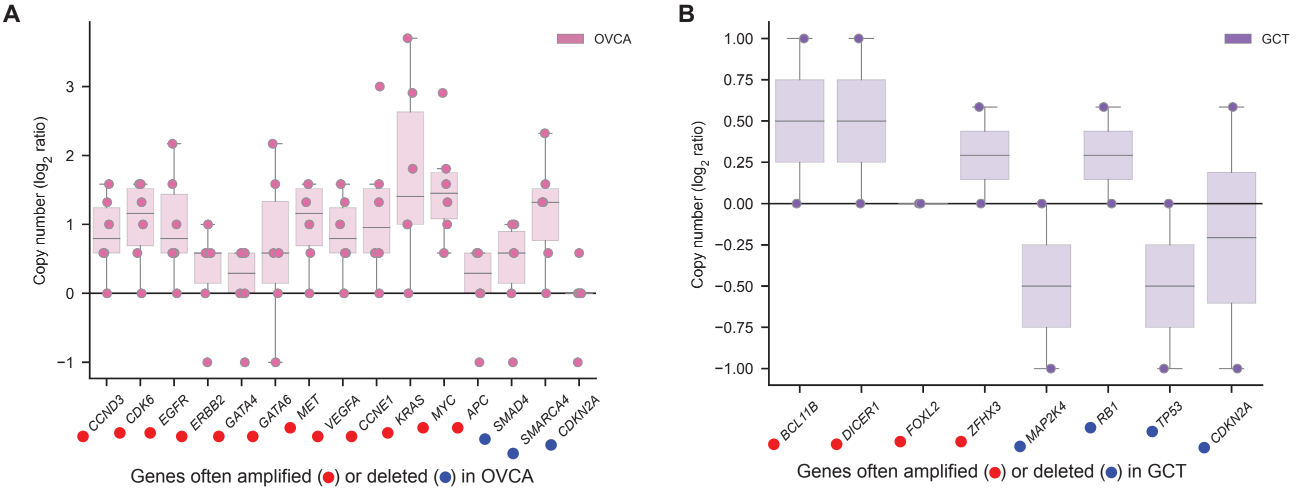

Supplemental Figure S5. NanoRCS copy number of genes often amplified and deleted per cancer type. Observed copy number distribution across the (A) OVCA (pink), (B) GCT (purple) samples in this study. Each data point represents a single sample. Commonly amplified (red) and deleted (blue) genes per cancer type are indicated below the graphs.
