# Supplementary material for: Nanopore-based consensus sequencing enables accurate multimodal tumor cell-free DNA profiling
Source: Genome Res. 2025 Apr;35(4):886–99. doi: 10.1101/gr.279144.124 (PMC12047234; doi:10.1101/gr.279144.124)
Supplement: Supplement 6 [file Supplemental_Fig_S6.pdf]

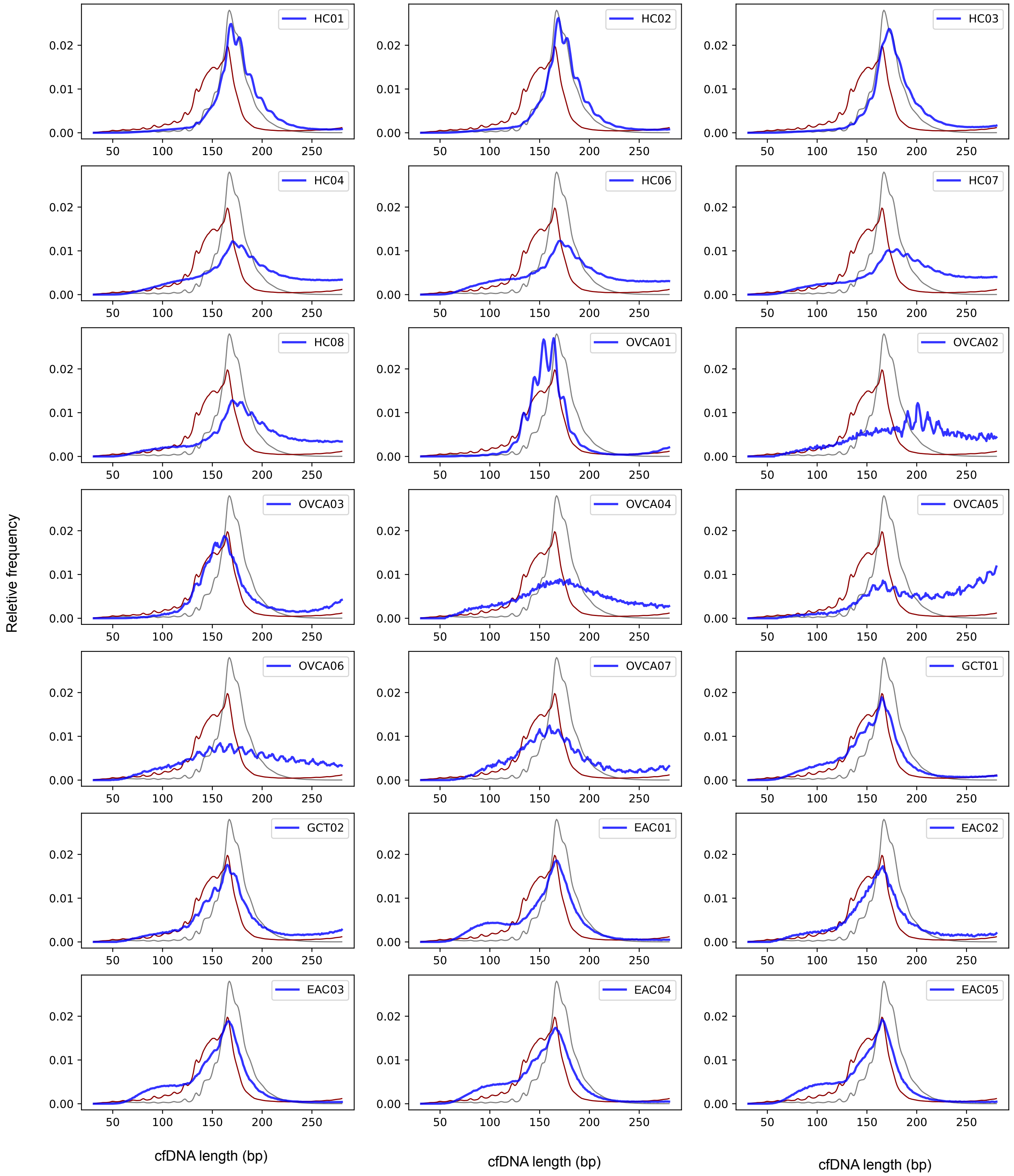

Supplemental Figure S6. NanoRCS cfDNA length profile compared to 2 signatures in NMF. Two representative NMF cfDNA length profiles adapted from Renaud et al. 2022. shown in Fig 4c are compared to all cfDNA samples observed with NanoRCS (normalized between 30-220 bp as described in Methods.) Signature 1, predominantly observed in healthy individuals, indicated in gray, and Signature 2, indicative of tumor-derived cfDNA, indicated in red. Blue lines represent observations through NanoRCS in the cfDNA. Sample names were indicated on the subplots; HC, healthy controls; OVCA, ovarian carcinoma; GCT, granulosa cell tumor; EAC, esophageal adenocarcinoma.
