# Supplementary material for: Nanopore-based consensus sequencing enables accurate multimodal tumor cell-free DNA profiling
Source: Genome Res. 2025 Apr;35(4):886–99. doi: 10.1101/gr.279144.124 (PMC12047234; doi:10.1101/gr.279144.124)
Supplement: Supplement 7 [file Supplemental_Fig_S7.pdf]

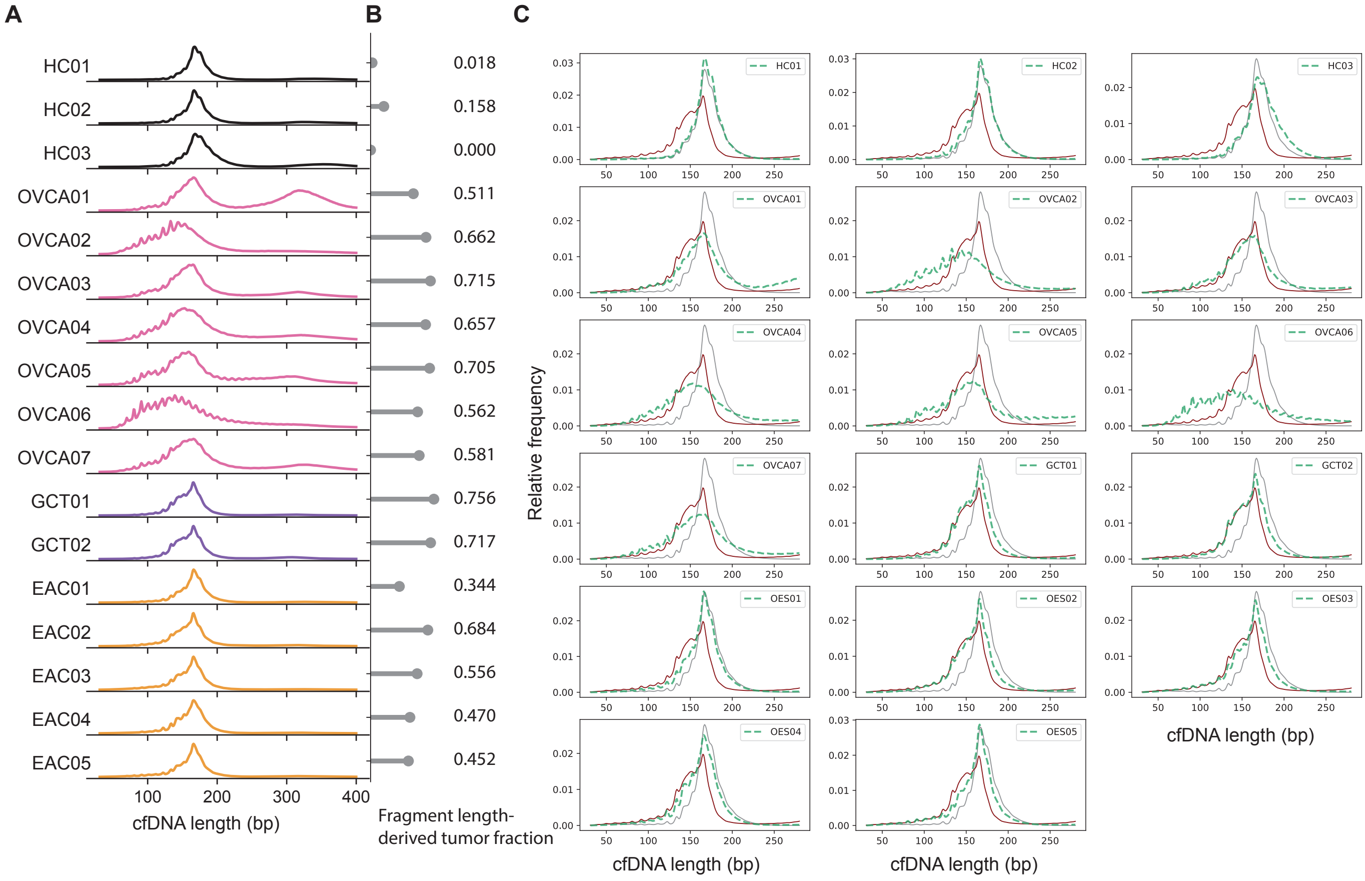

Supplemental Figure S7. NovaSeq cfDNA fragmentation length distribution and derived tumor fraction from NMF. (A) cfDNA fragmentation length profiles for each sample, categorized by sample type. HC, healthy controls; OVCA, ovarian carcinoma; GCT, granulosa cell tumor; EAC, esophageal adenocarcinoma. The profiles display the distribution of cfDNA fragment sizes ranging from 30-400 base pairs (bp). (B) Normalized non-negative matrix factorization (NMF)-derived tumor fraction (TF) for each sample. Each bar represents the proportion of the tumor-derived cfDNA component relative to the total cfDNA, with TF values annotated to the right of each bar. (C) cfDNA fragmentation length compared to 2 signatures derived from Renaud et al. 2022. Signature 1, predominantly observed in healthy individuals, indicated in gray, and Signature 2, indicative of tumor-derived cfDNA, indicated in red. Blue lines represent. Green lines represent observations through NovaSeq in the cfDNA.
