# Supplementary material for: Nanopore-based consensus sequencing enables accurate multimodal tumor cell-free DNA profiling
Source: Genome Res. 2025 Apr;35(4):886–99. doi: 10.1101/gr.279144.124 (PMC12047234; doi:10.1101/gr.279144.124)
Supplement: Supplement 8 [file Supplemental_Fig_S8.pdf]

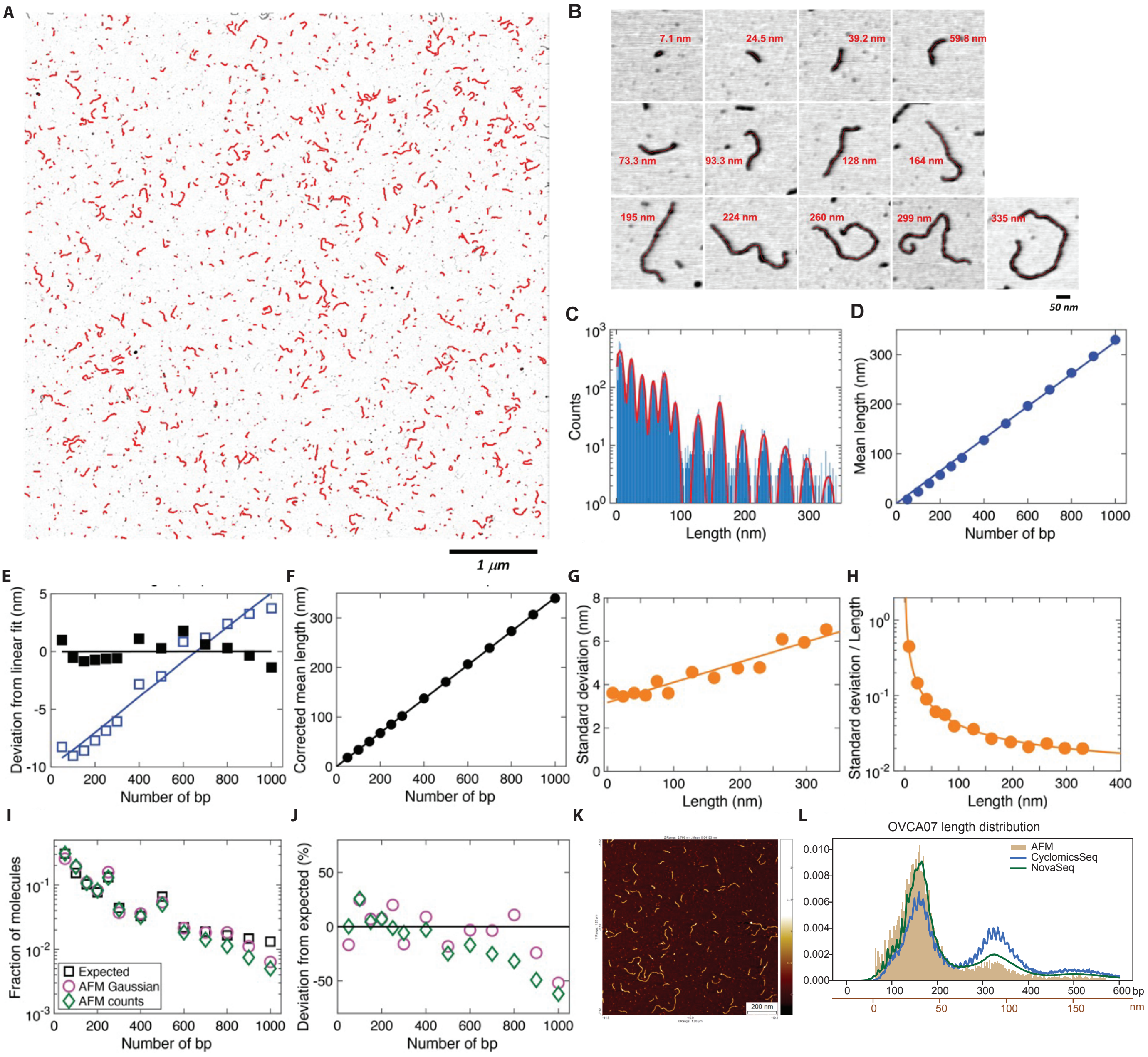

Supplemental Figure S8. Analysis of AFM data for the DNA ladder. (A) AFM images visualizing the Thermo Scientific GeneRuler 50 bp DNA Ladder control sample with fragments in the range of 50 bp to 1000 bp (50, 100, 150, 200, 250, 300, 400, 500, 600, 700, 800, 900, and 1000 bp). The DNA tracing is shown in red. The scale bar represents 1 um. (B) Zoom-in of the AFM image on 13 DNA fragments of different lengths from the DNA ladder sample. Red numbers indicate the length determined from DNA tracing for each molecule. (C) Histogram of DNA lengths from AFM images of the DNA ladder sample. DNA length values were determined by skeletonization (N = 13,970 molecules in total) from images as shown in panel a). 13 peaks are clearly visible in the histogram (blue data), corresponding to the 13 different DNA lengths present in the DNA ladder sample. The red line is a global fit of 13 Gaussians to the data. (D) Mean lengths determined from the Gaussian fits vs. number of base pairs. The line is a linear fit without y-offset and yields a slope of 0.326 nm/bp. While the data are well described by the linear dependence overall, we notice systematic deviations from linearity. In particular it is apparent that short lengths fall below the linear fit. (E) Deviations between the linear fit and the measured DNA length. Blue data points are for the length data shown in panel d). The solid blue line is a linear fit to the deviation data. Short DNA constructs appear too short relative to longer molecules. Black data points are for corrected length values with an overall additive offset of 10.0 nm. The solid black line is a fit for the black data points. (F) Corrected mean lengths determined from the Gaussian fits after including a constant

additive offset of 10.0 nm is plot against the number of base pairs in the expected DNA ladder fragments. The line is a linear fit without y-offset, yielding a slope of 0.341 nm/bp. From this, we derived the conversion Lbp=(Lnm+10.0)/0.341.(G) Standard deviations of the Gaussian fits vs. DNA length. The line is a linear fit to the data. The data are well described by a model that has a constant error of 3.2 nm and, in addition, a relative error of ~1% on the DNA length. (H) Standard deviations of the Gaussian fits normalized by DNA length vs. DNA length. These are the same data and model as in panel g); the normalization to length highlights that the constant relative error dominates at longer lengths, and the absolute error dominates small length measurements. (I) Fraction of the number of molecules in each "rung" of the DNA ladder. The black squares are data from the vendor's specifications. Magenta circles are data obtained from the areas of the fitted Gaussians in panel c). Green diamonds are data obtained from the counts in each peak, whereby the positions of minima between peaks define the borders between peaks. (J) Relative deviations between the fractions of molecules determined from AFM analysis (from the areas of the Gaussian in magenta or from the counts in each peak in green) and the expected fractions. It is apparent that the AFM analysis (using either quantification method) systematically under-counts the number of long molecules (> 800 bp). (K) AFM images visualizing OVCA07 cfDNA fragments (yellow). The scale bar represents 200 nm. (L) Density plots comparing the length distribution obtained from 3 different techniques (blue, NanoRCS; green, NovaSeq; brown, AFM) in sample OVCA07. X-axis in brown showing the length in nm for AFM data, X-axis in black showing the length in bp for sequencing techniques. The conversion between two axes are derived from the analysis on the ladder experiment in panel f, Lbp = (Lnm+10.0)/0.341.
