# Supplementary material for: Nanopore-based consensus sequencing enables accurate multimodal tumor cell-free DNA profiling
Source: Genome Res. 2025 Apr;35(4):886–99. doi: 10.1101/gr.279144.124 (PMC12047234; doi:10.1101/gr.279144.124)
Supplement: Supplement 9 [file Supplemental_Fig_S9.pdf]

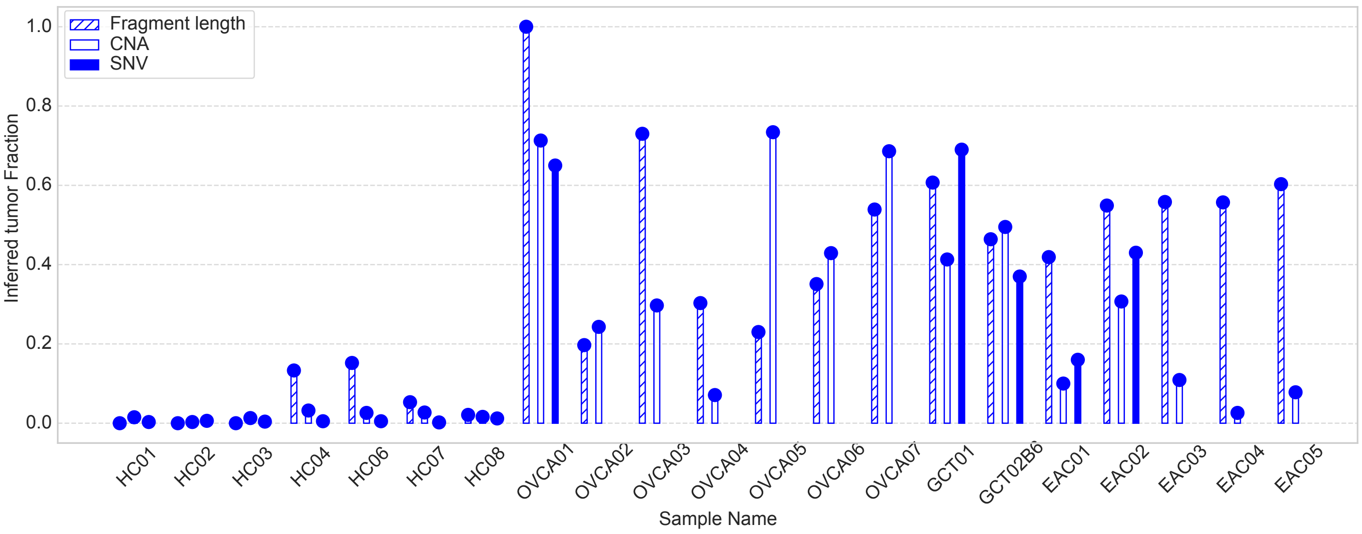

Supplemental Figure S9. Inferred tumor fraction in NanoRCS cfDNA sequencing SNV, CNA, and fragmentomics modalities. Lollipop plots represent sample's inferred tumor fraction. SNV (solid), CNA (nofill), and fragmentation length (striped) are compared. Healthy control SNV tumor fractions are averaged across all five comparisons with tumor samples with known tumor background.
