# Supplementary material for: Nanopore-based consensus sequencing enables accurate multimodal tumor cell-free DNA profiling
Source: Genome Res. 2025 Apr;35(4):886–99. doi: 10.1101/gr.279144.124 (PMC12047234; doi:10.1101/gr.279144.124)
Supplement: Supplement 10 [file Supplemental_Fig_S10.pdf]

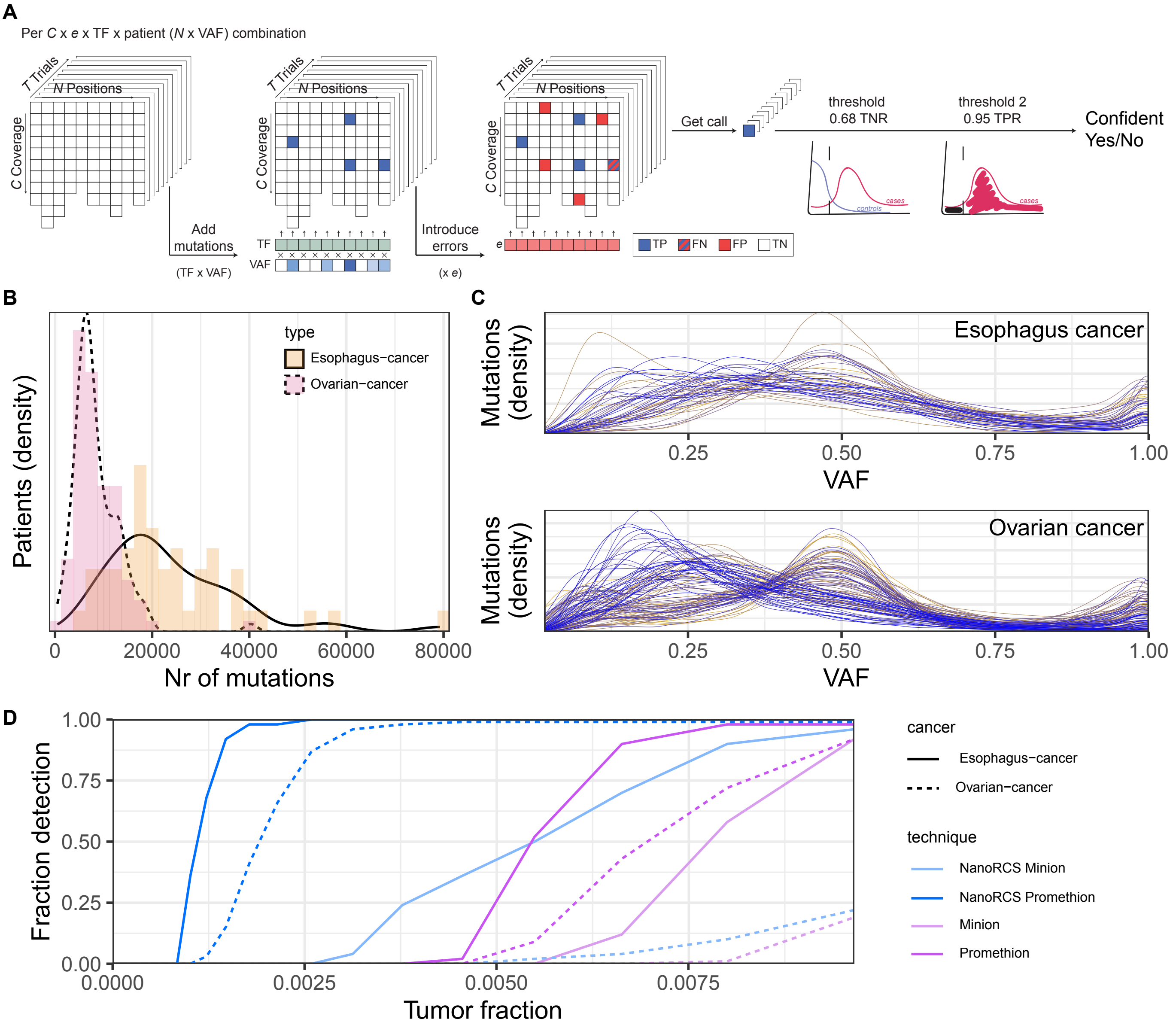

Supplemental Figure S10. Simulations to assess the lowest tumor fractions detectable for different platform throughputs (MinION vs PromethION) and with or without consensus calling for Esophagus and Ovarian cancer. (A) 10,000 T (Trials) in silico Nanopore and NanoRCS MinION and PromethION cfDNA sequencing runs were simulated for 50 EAC and 100 OVCA patients with N (number of observable positions; (depicted in (B) mutations at a mean C (coverage) of 0.04x, 0.8x, 0.25x and 3x for NanoRCS on Minion and Promethion, and native Nanopore sequencing on Minion and Promethion, respectively. Alternative allele observations were then added for 50 tumor fractions x VAF of the known mutations, depicted in (C). Finally, False positive and False negative observations were introduced for NanoRCS and native Nanopore sequencing based on the observed error rates depicted in Figure 2A. Confident detection was defined for a True negative rate of >0.68 and a True positive rate of >0.95, values in line with screening for MRD. (D) The fraction of tumor samples detectable at low tumor fractions for the four techniques. Colors indicate technique and linetypes represent the two different tumor types.
