# Supplementary material for: Nanopore-based consensus sequencing enables accurate multimodal tumor cell-free DNA profiling
Source: Genome Res. 2025 Apr;35(4):886–99. doi: 10.1101/gr.279144.124 (PMC12047234; doi:10.1101/gr.279144.124)
Supplement: Supplement 11 [file Supplemental_Fig_S11.pdf]

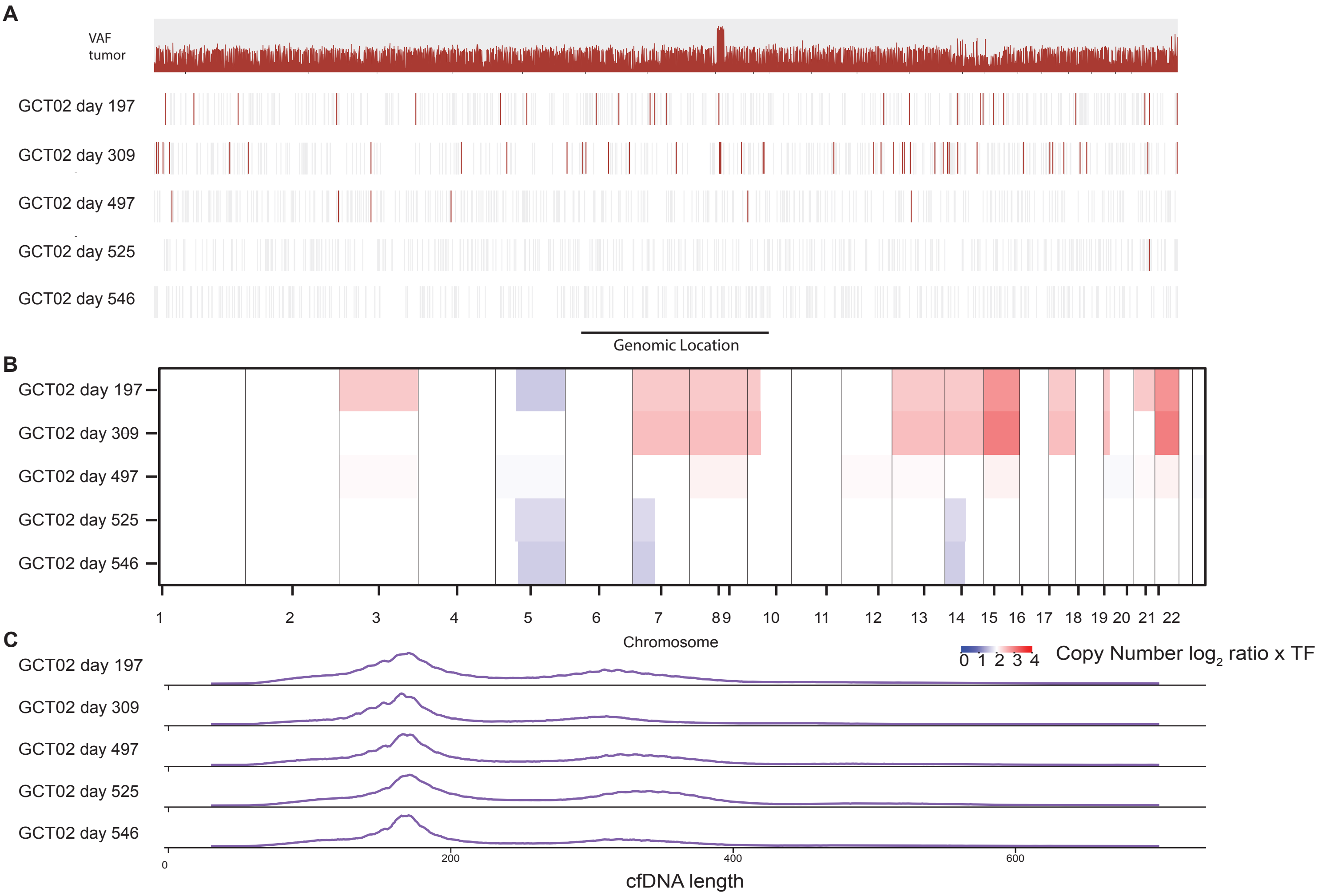

Supplemental Figure S11. NanoRCS genome-wide cfDNA sequencing of patient GCT02 during treatment. (A) SNV observations at all five measured time points. The top row shows the VAF of detected mutations in the tumor biopsy and the following five rows represent the MUT or REF allele observations in the liquid biopsies of patient GCT02. (B) Copy number alterations (CNAs) at all five measured time points. Red indicates copy number gain and blue indicates copy number loss, the color density indicates copy number $\log_2$ ratio multiplied by tumor fraction (TF, tumor fraction). Color intensity indicates the copy number alteration multiplied by the tumor fraction in cfDNA. (C) cfDNA fragmentation length profiles at all five measured time points. The profiles display the distribution of cfDNA fragment sizes ranging from 30-700 base pairs (bp).
