# Supplementary material for: Nanopore-based consensus sequencing enables accurate multimodal tumor cell-free DNA profiling
Source: Genome Res. 2025 Apr;35(4):886–99. doi: 10.1101/gr.279144.124 (PMC12047234; doi:10.1101/gr.279144.124)
Supplement: Supplement 12 [file Supplemental_Methods.docx]

# Supplemental Methods

#### cfDNA sample collection

Ascites of patients with ovarian cancer were collected, stored fresh frozen and processed as described in [(Kopper et al. 2019; Chris Jenske de Witte et al. 2020)](https://paperpile.com/c/nq5tBD/BUvbb+YLDCZ). The ascites samples used in this study were obtained from the HUB biobank (<https://huborganoids.nl/>). Plasma of patients with adult-type granulosa cell tumors was obtained and stored fresh frozen previously [(Groeneweg et al. 2021)](https://paperpile.com/c/nq5tBD/YIkVe). Blood of seven healthy donors with available whole genome sequencing data. For the healthy donors HC01, HC02, and HC03, the blood was taken and collected in 10 ml K2EDTA blood collection tubes (BD Vacutainer). The plasma was isolated immediately after blood collection by double-spin centrifugation. For healthy donors HC04, HC06, HC07 and HC08, the blood was collected in K2EDTA tubes and delivered within 24 hours. The plasma fraction was purified by double-spin centrifugation. The cfDNA was isolated from 0.5 to 10 ml of plasma using the Quick-cfDNA Serum & Plasma Kit centrifugation protocol according to the manufacturer’s protocol (Zymo Research). cfDNA was eluted twice in the provided elution buffer or ultrapure water and subsequently stored at −80 °C. The DNA was quantified using a Qubit fluorometer with double-stranded DNA (dsDNA) High Sensitivity Assay Kit (Thermo Fisher Scientific) and checked by digital electrophoresis using the TapeStation system (Agilent) using the High Sensitivity D1000 tapes. Blood, plasma, and tumors of patients with metastatic esophageal cancer were obtained, stored (fresh frozen and in FFPE, respectively) and processed as described previously [(van Velzen et al. 2022)](https://paperpile.com/c/nq5tBD/huCEU). The use of these human specimens for research purposes was approved by the Medical Ethics Committee of the UMC Utrecht (14-472 HUB-OVI, 17-868 and 20/055) and by the Medical Ethics Committee of the Amsterdam UMC (METC 2013_241), respectively. All participants provided written informed consent. Human specimens were stored at -80°C until further use.

Reference and tumor whole-genome sequencing data collection and preprocessing

Mapped sequencing data (BAM files) of reference blood and tumors from patients were collected from published articles for sample OVCA01, GCT01 and GCT02. For sample OVCA01, data obtained from fresh frozen biopsies was collected from [(Kopper et al. 2019; Chris Jenske de Witte et al. 2020)](https://paperpile.com/c/nq5tBD/BUvbb+YLDCZ). For patients with adult-type granulosa cell tumors, the BAM files obtained from fresh frozen biopsies were collected from [(Roze et al. 2020)](https://paperpile.com/c/nq5tBD/bz7Cv). Sequencing data of these samples can be found at the European Genome Archive (<https://ega-archive.org>) under accession numbers EGAS00001003073 and EGAS00001004249, respectively. BAM files of healthy controls were provided by the healthy controls. Since the sequencing data was mapped to different genome versions, BAM files were converted to FASTQ files and then remapped to hg37d5 with bwa mem (​​0.7.17-r1188) [(H. Li and Durbin 2009)](https://paperpile.com/c/nq5tBD/41oVm), for a uniform data analysis.

####

#### EAC reference and FFPE tumor sample whole-genome sequencing

DNA extraction from metastatic tumor tissue was performed for two EAC patients (EAC01, EAC02) from formalin-fixed paraffin-embedded (FFPE) slides of tumor tissue. DNA was extracted with QIAamp DNA FFPE Tissue Kit (Qiagen) according to manufacturer’s instructions, purified DNA was collected and stored in -80℃ until later use. Matched germline DNA and healthy control from peripheral blood mononuclear cells (PBMC) were collected and stored in -80°C until they were processed for DNA extraction. QIAamp DNA Blood Kits (Qiagen) was used to extract genomic DNA from PBMC. Both FFPE extracted DNA and PBMC DNA Libraries were prepared using KAPA HyperPlus Kit (Roche) following the recommended protocol. Libraries were run on a fragment analyzer to assess their size distribution. Libraries were pooled together based on expected final coverage and sequenced across multiple flow cell lanes. Whole genome sequencing was performed on the Illumina NovaSeq 6000 at 2 x 150 bp read length.

####

#### Somatic variant analysis in tumor biopsies

Whole genome sequencing (WGS) data of tumor biopsies and matched germline samples was mapped to the human reference genome (hs37d5). The WGS data underwent preprocessing steps using the Sarek [(Hanssen et al. 2023)](https://paperpile.com/c/nq5tBD/K5NOz) nf-core [(Ewels et al. 2020)](https://paperpile.com/c/nq5tBD/K6Aut) Nextflow pipeline (nf-core/sarek v3.1.2), adhering to the recommended GATK [(Van der Auwera and O’Connor 2020)](https://paperpile.com/c/nq5tBD/wECsT) best practices. Default options were activated, including: adapter trimming [(S. Chen 2023)](https://paperpile.com/c/nq5tBD/7YFk7) (fastp 0.23.2), alignment (bwa-mem, v0.7.17-r1188), MarkDuplicates (GATK4, v4.3.0.0), and Base quality score recalibration (GATK4, v4.3.0.0). Variant calling was performed subsequently by Strelka (v2.9.10) [(Saunders et al. 2012)](https://paperpile.com/c/nq5tBD/Ad9W6) and Mutect2 (GATK4, v4.3.0.0) with Sarek nf-core Nextflow pipeline by providing paired tumor and normal samples. Single-base somatic variants on autosomal chromosomes with a Strelka filter ‘PASS’ that intersect with Mutect2 results are selected. Subsequently, variants with filter label: “germline” and “panel of normal” were removed to remove potential false positive calls. For EAC samples, an extra step of selecting ‘PASS’ variants in Mutect2 was performed to reduce the amount of false positive calls. With these filtering criteria, a list of tumor-informed somatic SNV were recorded as a VCF file. These VCFs were used in the following Methods section: ‘Tumor-informed SNVs detection in NanoRCS’.

#### Library complexity estimation

An empirical Bayesian methodology by Daley et al.[(Daley and Smith 2013)](https://paperpile.com/c/q5kPHj/i7HO) is adopted to determine the molecular complexity of DNA sequencing libraries (shown in Suppl Figure 1). This technique evaluates the expected unique molecular content in a sequencing experiment. By considering a sequencing experiment as a random sampling from a DNA library, using initial shallow sequencing runs to gauge molecular diversity. Employing principles from capture-recapture statistics, it estimates the frequency of unique molecules, assisting in predicting the yield of additional reads. It uses rational function approximation to enhance accuracy, a technique often applied in theoretical physics. This approach enables precise predictions for sequencing datasets significantly larger than the initial sample.

#### NanoRCS library preparation of cfDNA detailed protocols

The NanoRCS protocol is divided into the following steps:

1. Preparation of the cfDNA for NanoRCS
2. Backbone-mediated circularization
3. Digestion of backbone concatemers
4. Removal of linear DNA
5. Rolling circle amplification
6. De-branching
7. Nanopore library preparation
8. **Preparation of the cfDNA for NanoRCS**

The cfDNA is prepared to ensure compatibility with the subsequent ligation step. Depending on the protocol variation, the cfDNA can be blunted or tailed, and it can be phosphorylated or de-phosphorylated (Table 1; Suppl. Table 1).

| **Protocol** | **cfDNA ends** | **cfDNA 5’** | **Backbone ends** | **Backbones 5’** |
| --- | --- | --- | --- | --- |
| A | blunt | de-phosphorylated | blunt | phosphorylated |
| B | blunt | phosphorylated | blunt | phosphorylated |
| C | G-tailed | phosphorylated | C-tailed | phosphorylated |

**Table 1. Protocol variations A-C for preparation of the cfDNA for NanoRCS.**

For a typical blunting reaction 5-50 ng of cfDNA are mixed with 5 𝜇l of CutSmart 10x (NEB), 5 𝜇l of dNTPs 1mM each, 2.5 𝜇l of DTT and 2 𝜇l of Blunting Enzyme (NEB) in a total reaction volume of 50 𝜇l. The reaction is incubated for 30 minutes at 22°C and heat inactivated at 70°C for 10 minutes. Optionally, the cfDNA can be de-phosphorylated by adding 6 𝜇l of Antarctic Phosphatase buffer 10x (NEB), 2 𝜇l of Antarctic Phosphatase Enzyme (NEB), and 2 𝜇l of water. The reaction is incubated at 37°C for 30 minutes and heat-inactivated at 70°C for 10 minutes. Once blunted, the cfDNA may undergo tailing, using DNA Pol I, Large (Klenow) fragment without exonuclease activity (exo-) (NEB) in combination with dATP or dGTP. The backbone is tailed in a similar fashion to the complementary nucleotide. The cfDNA is then circularized with the help of a backbone during step 2. The reaction is supplemented with three molar excess (relative to the initial amount of cfDNA) of backbone, 18 𝜇l of water, 5 𝜇l of CutSmart buffer 10x (NEB), 5 𝜇l of ATP 10 mM,, and 2 𝜇l of T4 DNA Ligase. The reaction is then incubated at 22°C for 4h and heat-inactivated at 70°C for 30 minutes. When blunt-ended cfDNA and backbone are used during the circularization, the backbone can self-circularize. To remove this byproduct, a Cas9 nuclease digestion is performed. The Cas9 enzyme is loaded with an RNA guide complementary to a sequence formed upon self-circularization of a backbone. In step 3, any residual linear DNA is digested using Exonuclease V (NEB). At this point, only circular BB-cfDNA molecules and original circular-cfDNA molecules are left and will serve as a template for the RCA. During RCA, long concatemers formed by repeats of the circular template are formed. The concatemers are further treated with T7 Endonuclease (NEB) to remove branches before being prepped for Nanopore sequencing.

1. **Backbone-mediated circularization.** The reaction is supplemented with 3 molar excess (relative to the initial amount of cfDNA) of backbone, 18 𝜇l of water, 5 𝜇l of CutSmart buffer 10x (NEB), 5 𝜇l of ATP 10 mM, and 2 𝜇l of T4 DNA Ligase. The reaction is incubated at 22°C for 4 hours and heat-inactivated at 70°C for 30 minutes.
2. **Digestion of backbone concatemers.** 1 𝜇l of Cas9 nuclease (NEB) and 2 𝜇l of single-guide RNA (check concentration) are added and incubated at 22°C for 30 minutes, then at 37°C for 1 hour. Since Cas9 binding to the DNA can interfere with the next step, the reaction is treated with 2 𝜇l of Proteinase K (NEB). The digestion is carried on at 37°C for 30 minutes, followed by heat inactivation at 98°C for 10 minutes.
3. **Removal of linear DNA.** The reaction is supplemented with 2 𝜇l Exonuclease V (NEB) and 5 𝜇l of ATP 10 mM, then incubated at 37°C for 30 minutes, then heat-inactivated at 70°C for 30 minutes.
4. **Rolling Circle Amplification.** Firstly, short random primers are annealed to the template. In particular, 1 𝜇l of exo-resistant RND primers 500 𝜇M (ThermoFisher) are added, the reaction is heated up to 98°C and then let cool down at room temperature. Next, the components for the RCA reaction are added: 22 𝜇l of water, 5 𝜇l of CutSmart 10x (NEB), 2 𝜇l of BSA (NEB), 15 𝜇l of dNTPs 10 mM, 4 𝜇l of Inorganic Pyrophosphatase (NEB) and 2 𝜇l of Phi29 DNA Polymerase (NEB). The reaction is incubated for 8 hours at 30°C.
5. **De-branching.** The RCA reaction was heat-inactivated at 70°C for 10 minutes, and then 2 𝜇l of T7 Endonuclease (NEB) was added. The reaction was incubated at 37°C for 30 minutes, purified with 75 𝜇l of AMPure XP beads, and eluted with 60ul of H_2_O, pre-warmed at 65°C.
6. **Nanopore Library Preparation.** Sequencing libraries were prepared according to the manufacturer's protocol version SQK-LSK109 (for R9.4 flow cells) and SQK-LSK114 (for R10.4 flow cells) using 1500 ng of purified RCA product as input DNA. For barcoding, the Native Barcoding Expansion Kit EXP-NBD109/EXP-NBD114 was used according to the manufacturer's protocol.

#### Somatic copy number alteration analysis and tumor fraction inference

We use ichorCNA [(Adalsteinsson et al. 2017)](https://paperpile.com/c/q5kPHj/Wsvc) software (commit 5bfc03e) to calculate the copy number alterations in all samples. The snakemake pipeline provided is used to perform HMMcopy (version 0.1.1) readCounter to acquire coverage per 1Mb bin and ichorCNA. We modified default settings to allow flexible search for high ploidy (CN=2,3,4) and high copy number (maxCN=8) solutions for all samples and report only chr1-chr22. For samples with inferred tumor fraction below 0.03, we performed another estimation with MRD settings upon the suggestions of the authors on parameter tuning and settings on low tumor fraction samples Namely, non-tumor fraction parameter restart values were set to lower, and no subclonal status is allowed, with maxCN=3. Detailed parameters provided to the snakemake pipeline can be found in Suppl. Table 5. Panel of normal (PoN) files used for NanoRCS runs and tissue biopsy sequencing are constructed with createPanelOfNormals.R. Sequencing of 3 HCs cfDNA and healthy blood cells in this study were used to construct the PoN of NanoRCS and tissue Illumina sequencing respectively. For Illumina NovaSeq runs, PoN provided by authors with ichorCNA software was used. IchorCNA provides several solutions of copy number alterations and derived tumor fraction sorted by the final log-likelihood of the EM convergence. The author stated in the wiki, “*Sometimes ichorCNA will choose a suboptimal solution. Some easy ways to spot a potentially incorrect solution are (1) A large proportion of CNAs are being called subclonal. (2) The majority of data points are brown or red, suggesting a whole genome amplification event. (3) There are two distinct copy number levels being called neutral.”* For low tumor fraction samples (tf = 0.03-0.1), they stated that tumor fraction estimate is most accurate when there is at least one amplification and deletion event spanning more than 100 Mb each. (<https://github.com/broadinstitute/ichorCNA/wiki/Interpreting-ichorCNA-results>) We used these principles plus the prior knowledge of ploidy of tumor tissue samples to select most likely correct solution for all samples, and the selected solution is provided in Suppl. Tables 6.

#### Atomic Force Microscopy imaging and sample preparation

cfDNA samples and a commercial DNA ladder (Thermo Scientific GeneRuler 50 bp DNA Ladder) were diluted to final DNA concentration of 0.5 ng/mL in buffer of physiological ionic strength at room temperature (10 mM Tris-HCl; pH 7.0; 200 mM NaCl). DNA solutions were deposited by drop-casting a volume of 10 𝜇L (HC03) or 20 𝜇L (DNA ladder, OVCA01, OVCA07) onto poly-L-lysine for 30 seconds, followed by rinsing with 20 mL of milliQ water, and finally dried using a gentle flow of filtered Argon gas [(Vanderlinden et al. 2014)](https://paperpile.com/c/q5kPHj/NmF5). The dried sample was immediately measured in air using a Nanowizard Ultraspeed 2 AFM (Bruker), equipped with a high-speed Z-scanner and using a FastscanA cantilever in high-speed tapping mode. A total of 6-11 images of 6 𝜇m x 6 𝜇m were acquired with 4096 x 4096 pixels at a line rate of 3 Hz for each sample.

#### Atomic Force Microscopy (AFM) image analysis for cell-free DNA length determination — analysis of length accuracy and portion distribution

To test the accuracy and precision of our AFM-based DNA length measurements, we analyzed a DNA ladder sample with fragments of length 50, 100, 150, 200, 250, 300, 400, 500, 600, 700, 800, 900, and 1000bp (Suppl. Fig. 7A-J). Our tracing by skeletonization results in a DNA length distribution for the DNA ladder sample that clearly resolves all 13 fragments in the range from 50 to 1000 bp. The mean lengths determined from the center positions of the Gaussian peaks increase linearly with DNA length in bp (Suppl. Fig. 7C) and the fitted slope of 0.326 nm/bp is in line with previous reports of DNA length from AFM imaging [(Konrad et al. 2021a)](https://paperpile.com/c/q5kPHj/3WeQ). Nonetheless, it is noticeable that data points at short lengths fall below the linear fit line, which is readily apparent in a plot of the deviations from linearity (Suppl. Fig. 7D-E), which show small but systematic deviations, with short lengths falling below the fitted constant slope and long lengths above it. These deviations likely stem from the fact that tracing by skeletonization misses some length at the ends of the molecules, which introduces an overall constant offset to the length measurements (since all traced molecules have two ends). We therefore correct the data by adding a constant offset for all values (Suppl. Fig. 7F).

We exploit the fact that the DNA ladder contains DNA fragments in known proportions to test how well our AFM analysis reproduces the expected proportions based on the vendor’s specifications. Overall, the fractions of molecules determined from AFM analyses closely track the expected values, despite the large variation in the number of molecules in each band (Suppl. Fig. 7I-J). The mean (absolute) deviation for DNA lengths < 800 bp is 12%. For long molecules (> 800 bp) the AFM analysis systematically undercounts the number of molecules, which is likely due to the fact that our skeletonization approach excludes strands that cross or self-overlap from further analysis, since the probability to overlap and thus be excluded from further analysis increases with DNA length [(Shore et al. 1981; Konrad et al. 2021b)](https://paperpile.com/c/q5kPHj/7bi0+SwjG).

#### Simulation of real time tumor-informed SNVs detection with NanoRCS

To determine the time required to detect a mutant allele through real-time sequencing across various tumor fractions and tumor-informed SNV counts, we conducted simulations based on the observed real-time throughput of six PromethION runs in this study, including 1% and 0.5% OVCA01 samples from an admixture experiment, GCT02 day 525 and day 546, HC01, and HC02. The "Basecalled Reads Passed" metrics at 5-minute intervals were extracted from the sequencing summary files of these runs, with 50% of the reads considered as contributing to unique consensus reads (Suppl Fig. 3A). The read length of each read was simulated based on the average read length distribution derived from the mean of all samples in this study, and by summing up all read lengths, total cfDNA bases per time interval is calculated. A fraction of the sequence genome span is composed of tumor-informed SNV counts (for example, 6000 and 15000 SNVs with VAF = 0.5 for all variants as shown in Suppl. Fig. 3B-C). By the total cfDNA bases, the tumor-informed SNV fraction in genome, VAF of all variants, and tumor fractions, we calculate the number of mutant alleles detected across different tumor fractions over a 360 minute time period for each tumor fraction. The background error rate is modeled similarly, by multiplying the total cfDNA bases to the NanoRCS error rate instead of the tumor fraction. The earliest time point of tumor-specific SNV detection was determined by identifying the first value that is above the background errors in the dataset.

#### Calculation of minimal error rate for an uninformed approach

With a sequencing error rate of Q40 (which is higher than most of the current techniques, including NanoRCS) and a mutational load of 20,000 (which is quite high; see Suppl. Fig 9), there is a sequencing error every 10,000 base pairs (for 1x coverage) and a true mutation is present every 160,000 base pairs. This means that with the current sequencing techniques, the error rate is at least 1,600x higher (16 times + 1%TF) than the mutational load in a 1% tumor sample sequenced 1x. We think a Q60 (error every 1,000,000) may be sufficient for an uninformed approach for detecting tumor fractions as low as 1%, with an error rate 1.6x higher than the mutation rate. This means you will observe 2x more ‘mutations’ in a tumor sample compared to a HC.
