# Supplementary figures and images for: Nanopore-based consensus sequencing enables accurate multimodal tumor cell-free DNA profiling
Source: Genome Res. 2025 Apr;35(4):886–99. doi: 10.1101/gr.279144.124 (PMC12047234; doi:10.1101/gr.279144.124)

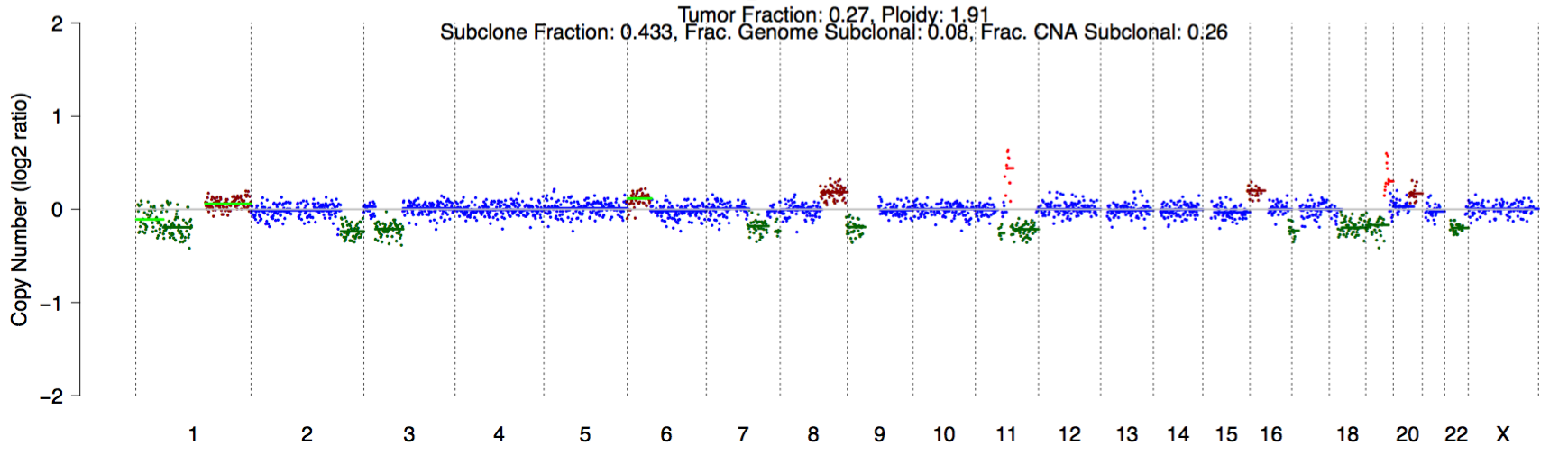

Supplement: Supplement 13 [file Supplemental_Code.zip › Supplemental_Code/NanoRCS/05_cna/ichorCNA/scripts/example_gw_plot.png]
